# How to determine hands’ vibration perception thresholds – a systematic review

**DOI:** 10.3758/s13428-024-02534-w

**Published:** 2024-12-28

**Authors:** Emanuel Silva, Isabel C. Lisboa, Nélson Costa

**Affiliations:** 1 Algoritmi Research Centre, University of Minho, Campus de Azurém, 4800-058 Guimarães, Portugal; 2 NOVA School of Science and Technology, UNINOVA-CTS and LASI, NOVA University Lisbon, 1099-085 Lisbon, Portugal

**Keywords:** Vibration perception threshold, VPT, Systematic review, Hands, Fingers

## Abstract

The vibration perception threshold (VPT) is the minimum amplitude required for conscious vibration perception. VPT assessments are essential in medical diagnostics, safety, and human–machine interaction technologies. However, factors like age, health conditions, and external variables affect VPTs. Various methodologies and distinct procedures have been used to access VPTs, leading to challenges in establishing standardized protocols. Following the PRISMA methodology, this systematic review was conducted to answer the research question: “How are vibration perception thresholds assessed on the glabrous skin of the hands and fingers of healthy humans?” Searches were conducted across five databases to locate recent studies reporting data from VPT assessments, published in English, between 2012 and 2023. Thirty-nine studies met the inclusion criteria. Data on study goals and various methodological aspects was categorized and analyzed. Information gaps were identified, and this review offers recommendations for future studies to enhance standardization and facilitate data comparison. This review also suggests directions for future research, aiming to improve our understanding of how humans perceive haptic information.

**Supplementary Information:**

The online version contains supplementary material available at 10.3758/s13428-024-02534-w.

## Introduction

Humans interact through touch with many objects every day. Our sense of touch can collect a lot of information from the simplest of actions, be it the simple and familiar contact of our clothes with our bodies, leaning against a wall, or grabbing an object. Our brain constantly receives information regarding the surfaces we touch, from how rough they are, how hot or cold, or even if they vibrate slightly, strongly, or not at all, among many others. All these data are first processed subconsciously, and if our brain deems any of it to be important enough—to warn us of danger or to transmit other noteworthy information, for example—it gets transmitted to our conscious mind so that we can perceive it (Jones & Lederman, 2006).

Out of the entire surface of our bodies, our sense of touch is most sensitive at our hands and fingers (Jones & Lederman, 2006). Despite extensive research, however, there is still a lot that we do not know about the fundamental neurophysiology and foundations of the human perception of touch. Currently, the four-channel theory is the most well-known and accepted framework regarding the mechanisms of vibrotactile perception on the glabrous (non-hairy) human skin. This theory states that four populations of tactile afferents, each ending in one of four types of mechanoreceptors, are located under the surface of glabrous skin (Bolanowski et al., 1988; Jones & Lederman, 2006). These mechanoreceptors are each attuned to sensing and responding to a certain type of haptic stimuli, with two types, in particular, being specialized towards sensing vibrations (Basdogan et al., 2020): (1) the Meissner corpuscle fibers, thought to be sensitive to frequencies in the 10–100-Hz range (Labs et al., 1978, as cited in Bolanowski et al., 1988;  Gescheider et al., 1985, as cited in Bolanowski et al., 1988; Verrilo & Bolanowski, 1986, as cited in Bolanowski et al., 1988), and maximally sensitive between 5 and 40 Hz (Talbot et al., 1968); and (2) the Pacinian corpuscle fibers, thought to be sensitive to frequencies between 40 and 800 Hz, and maximally sensitive around 300 Hz (Verrillo, 1975, as cited in Bolanowski et al., 1988). However, even if a vibration of a given frequency is sensed by a mechanoreceptor, we will not consciously perceive it unless its amplitude is high enough.

The amplitude of a vibration dictates how “strong” said vibration is, with higher-amplitude vibrations being easier to perceive than those with smaller amplitudes. In psychophysics—the field dedicated to the study and understanding of the relation between the physical characteristics of a stimulus and the sensations it produces—the smallest amount of stimulus energy that is required to produce a noticeable sensation is generally called the *absolute* or *stimulus threshold* (Gescheider, 2015). Various methodologies (and respective variations) have been developed in this field to study the thresholds for various sensations, such as sound, vision, and touch. As a result, researchers have a wide range of options when selecting which psychophysical method to use in their studies (Gescheider, 2015; Siao & Cros, 2003).

In the field of study of touch perception, the term vibration perception threshold—or vibrotactile perception threshold, among others—(VPT) is more commonly used than the terms absolute or stimulus threshold to particularly refer to the minimum amount of amplitude needed for a vibration of a certain frequency to be perceived (Gescheider, 2015; Griffin, 1990; Morioka & Griffin, 2002). VPTs are commonly the main dependent variable in psychophysics studies focused on the perception of vibrations. However, VPTs are affected by other factors besides frequency, both external factors (e.g., surrounding temperature, humidity, or the size of the point of contact) and factors internal to the human body (e.g., a person’s age, overall health, or the presence of various neuropathies). For more information regarding factors known or suspected to affect VPTs, please refer to the works of Gandhi et al. (2011), Griffin (2012), and Silva et al. (2024).

The presence of neuropathies, in particular, has fuelled various studies assessing VPTs, as neuropathies such as diabetic neuropathy and diabetic foot ulcer, among many others, are known to impair thresholds at the fingertips/pads and the sole of the feet at their early stages (Ising et al., 2018; Lindholm et al., 2019). Thus, to aid with diagnostical procedures or various pathologies, medical professionals may collect VPT data from patients to compare their results against normative VPT data assessed from healthy subjects. If the results from these patients deviate from this normative data (e.g., if the VPTs from the patients are significantly higher than those in the normative dataset, indicating a reduced sensibility), this information, in combination with information from other medical exams, can be used by medical professionals to narrow down on a diagnosis.

VPTs are not just useful in the medical field, and even in this field, they are not just used for diagnostical purposes. Information about VPTs, for example, can be used to complement communications with human–machine interfaces (HMIs). For instance, researchers have studied how vibrations can be used to improve the information provided by surgical machines, such as those used to conduct surgeries remotely (Bark et al., 2013). Other researchers, in turn, have studied how vibrotactile feedback could be used in devices that promote social interaction between users, such as through a haptic glove (Krishna et al., 2010) or a device that emulates social touch (Rantala et al., 2011), while others have looked at vibrotactile feedback as an alternative way to provide information to motorcycle riders to promote their safety (Lisboa et al., 2024). Still, one key factor is that, for vibrations to be useful in HMIs, they must be perceptible to users over as many interaction conditions and scenarios as possible, such as when users are holding the device with a tight or gentle grip, or when they are using it in a noisy environment (Schneider et al., 2017).

To increase the chances of vibrotactile feedback being perceived over as many conditions as possible, developers might set vibrations to always actuate at their maximum amplitude. However, this might alienate users who might feel that said high amplitudes are uncomfortable, annoying, or distracting. Furthermore, this also greatly limits the potential use of vibrations in human–machine communication. By manipulating the parameters that define a vibration, such as its frequency, tempo, and/or amplitude, vibrotactile feedback can be pushed beyond its classical use of being turned on and off. For example, vibrations with the same frequency but with different amplitude magnitudes could be used to inform users regarding the various levels of urgency of incoming email notifications (Culbertson et al., 2018). However, if developers always set the amplitudes of their vibrations to their maximum magnitude, they are not taking advantage of the vibrotactile signal on its full potential. Thus, developers should make use of VPT information to design vibrations that are perceptible to users without being uncomfortable. At later stages of the product’s development, VPT information could further be combined with information regarding other psychophysical metrics, such as the just-noticeable difference (JND)—i.e., the minimum amount of magnitude difference required for two similar stimuli to be perceived as being different (Kingdom & Prins, 2016)—to further push the role of vibrotactile feedback in human–computer communication.

That said, due to the multitude of factors that are known or are suspected to affect vibration perception, data from VPT assessments is not a constant “one size fits all,” and may instead require adjustments and further assessments based on the user, their characteristics, their environment, and their use-cases, to more appropriately respond to their needs. On the other hand, it may be difficult for both researchers and designers to decide which assessment methods are better suited for their goals, due, in part, to the multitude of methods that are available for this, but also due to the many works that have assessed VPTs for various ends, some of which may even overlap with their objectives.

For these reasons, the International Organization for Standardization (ISO) created a set of ISO Standards, to both standardize VPT assessments, and also provide a set of normative data, collected from healthy individuals, for some frequencies (International Organization for Standardization, 2003). These ISOs include, among other information, details with instrumentation specifications, methodologies to use, and how to report results (International Organization for Standardization, 2001). However, and most importantly, the normative data set that is available is far from being complete. As mentioned previously, VPTs are affected by many physiological, neurological, and environmental factors, making it challenging to capture and/or control for the full spectrum of variables that contribute to VPTs. This might result in a limited representation of the available normative data that may not accurately represent other populations with distinctive characteristics; and/or may not generalize to other environmental settings. Thus, it is critical to improve these normative datasets, making them more robust and flexible across various populations, pathologies, and environments.

In summary, given their current—and future—usefulness, VPTs are already the focus of many research studies. One of the ways in which VPT data can be provided to researchers and developers is in the form of normative data. However, due to the myriad factors affecting VPTs, it is difficult to compile a single normative dataset that encompasses all these variables. Furthermore, their influence is normally only assessed because of a study’s specific goals, and thus, data gathered from specific frequencies, or from certain population groups, may be minimal or non-existent. This means that if researchers and developers intend to use VPT data, they must first scour through the multitude of VPT studies that have already been conducted to see if the variables they require have already been studied. If not, then they will have to collect said data themselves, which means deciding on which methods to use based on the body of work of the studies that came before.

To aid in this matter, this review intends to investigate the experimental methodologies of numerous studies that have conducted VPT assessments on the glabrous skin of the hands and fingers of healthy subjects in recent years, to organize, catalog, and analyze their characteristics. The goal is to provide an overview of what has been studied before and how. Moreover, we intend to provide a general idea about some of the variables that have been less explored regarding VPTs, such as less studied frequencies and hand locations. Thus, this review intends to answer the following research question: “How are vibration perception thresholds assessed on the glabrous skin of the hands and fingers of healthy humans?”.

To this end, we conducted a systematic review using the Preferred Reporting Items for Systematic reviews and Meta-Analyses 2020 (PRISMA 2020) methodology (Page et al., 2021), identifying 1022 relatively recent records, published between January of 2012 and December of 2023, of which 39 studies were included for review. We present and analyze the content of these 39 articles, outlining their main goals and exploring and discussing the methods used to measure vibration perception thresholds (VPTs) in the glabrous skin of the hands and fingers.

## Methods

### Search methods to identify studies

This paper focuses on the experimental protocols of studies conducted with healthy human subjects. The aim is to categorize and systematically review the available experimental protocols that have been used to assess VPTs at the locations of interest to this review—namely on the glabrous skin of the hands and fingers. These locations were selected for two main reasons: (1) those are the areas that are most sensitive to tactile sensations, and (2) they are most often used to interact with haptic objects, including HMI devices. We only focused on studies involving healthy subjects because these are the most common throughout the literature, with said subjects being either the main subjects of a study or acting as comparison subjects.

The PRISMA 2020 methodology (Page et al., 2021) was followed throughout this review. The acquisition of studies and subsequent analysis of title, abstract, and content, were conducted by a singular author (ES, first author). Studies were acquired through searches in five of the leading electronic databases, namely Web of Science, Scopus, PubMed, IEEE Xplore, and the ACM Digital Library. The employed search terms were “vibration”, “perception”, “threshold”, “hand”, and “finger.” Search strings were created using these terms and the Boolean operators “AND” and “OR” and were adapted as needed to better suit the interface of each database.

Searches were conducted on three different occasions. An initial search (henceforth referred to as “2012–2022 search”) was performed by January 13, 2023, restricting results to only those published in the English language between January 1, 2012, and December 31, 2022. This search was conducted only on the Web of Science, Scopus, and PubMed databases. While research on the human perception of vibration has been published for many years (for examples, see Bolanowski et al., 1988; Geldard, 1940; and Johansson & Vallbo, 1979), at the time when the “2012–2022 search” was completed, our goal was only to review papers published over the previous 10 years, to keep the focus only on relatively recent research studies—for readers interested in the methodological aspects of research studies that assessed VPTs and which were published before 2012, we suggest the work of Gandhi et al. (2011), whose comprehensive review on this topic served as one of the main inspirations for this review. However, as the analysis process took longer than initially planned, we decided to further include papers published during 2023. Thus, a second search (henceforth referred to as “2023 search”) was carried out by November 24, 2023, restricting results to only those published in the English language between January 1, 2023, and December 31, 2023. This search was also only conducted on the Web of Science, Scopus, and PubMed databases. A third search (henceforth referred to as “2012–2023 search”) was further conducted by May 20, 2024, to include results from the IEEE Xplore and ACM Digital Library databases, published between January 1, 2012, and December 31, 2023. The results of this search were restricted to only those published in the English language between January 1, 2012, and December 31, 2023. The final search strings used in each database on each occasion, as well as the number of entries returned in each one, are available as supplementary material.

**Fig. 1:**
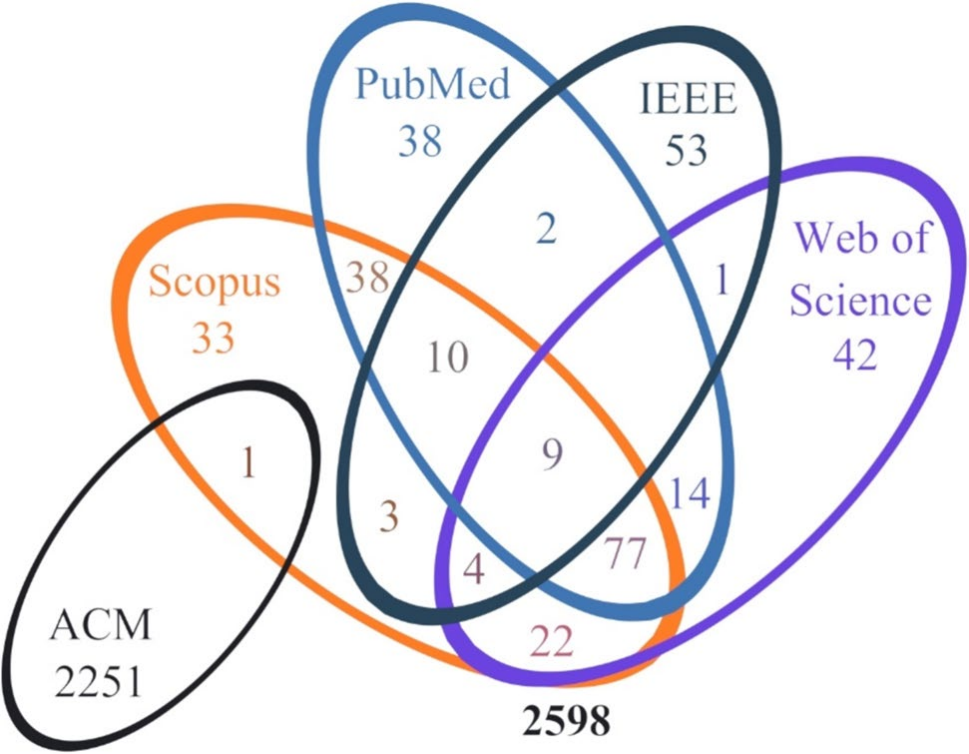
Number of records found across all searches after duplicates were removed

A total of 3396 records were identified from across all 24 searches. Of these, 3285 entries were exported, using each database’s exportation tools, to Microsoft® Excel® for Microsoft 365 MSO (version 2210), while 111 entries could not be exported due to database limitations. All 3285 records were included in a single Excel file, where duplicates were screened for and removed. At the end of this process, 2598 records were identified. A visual representation of the distribution of these 2598 records across all five databases is presented in Fig. 1.

### Eligibility criteria – Screening title and abstracts

Records were systematically screened for retrieval based on the information presented in their title and abstract, according to the following criteria:

The title and/or abstract informs that:

1. *Abstract.VPT* (*A.VPT*) — The paper will report the results of at least one quantitative experimental study in which VPT assessments were made, either as the main goal of the study, or as one of the study’s metrics.
2. *Abstract.Hand/Finger* (*A.Hand/Finger*) — At least one of the locations where VPTs were assessed was at the glabrous skin of a hand or finger.
3. *Abstract.Healthy/Control* (*A.Healthy/Control*) — At least one study or experiment was conducted with a group of healthy or control individuals of any age.

These criteria were assessed for each record as being either present ("Yes"), absent ("No"), or not mentioned or not overtly clear ("N/M"). All records were then organized into inclusion (Abstract Inclusion) and exclusion (Abstract Exclusion) groups. Afterward, records on the Abstract Inclusion were further divided into four sub-groups based on the number of "Yes" and "N/M" attributed to each record (see Table 1). Records with "Yes" on all three criteria were included in the "Abstract Inclusion 3" group; records with “yes” on two criteria and one criterion not overtly clear ("N/M"), in the “Abstract Inclusion 2”; records with “yes” in only one criterion in the “Abstract Inclusion 1” and papers with "N/M" on all three criteria were included in the "Abstract Inclusion 0".

**Table 1:** Title/Abstract inclusion and exclusion groups with the number of records attributed to each

Group		No. included
Abstract Inclusion 3	3 "Yes", 0 "N/M"	53
Abstract Inclusion 2	2 "Yes", 1 "N/M"	63
Abstract Inclusion 1	1 "Yes", 2 "N/M"	38
Abstract Inclusion 0	0 "Yes", 3 "N/M"	61
Total included for retrieval		215
Abstract Exclusion 1	≥ 1 "No"	1984
Abstract Exclusion 2	Literature review or presentation/description of instruments/tools; Records that were not papers	379
Total excluded from retrieval		2363

*N/M* not mentioned or not overtly clear

Records with a "No" on any criteria were included in the "Abstract Exclusion 1" group. Records of other literature reviews or presentations/descriptions of new assessment instruments or tools that did not contain information regarding a quantitative experimental study were grouped into a separate category, "Abstract Exclusion 2", as they were beyond the scope of the present review. Table 1 presents all the abstract inclusion and exclusion groups, accompanied by the number of records included in each. In total, 215 reports were sought for retrieval.

### Eligibility criteria – Content

Of the 215 reports sought for retrieval, four could not be retrieved due to lack of access. Thus, the content of the 211 retrieved reports was manually analyzed for eligibility according to five criteria:

1. *Content.VPT* (*C.VPT)* – The paper reports on the result(s) of at least one quantitative experimental study that assessed the VPT of a given frequency or similar procedure (e.g., the lowest amount of amplitude reached before a vibration of a given frequency is no longer detected, i.e., vibration extinction/disappearance threshold; the lowest amount of frequency reached before a vibration of a given amplitude is no longer detected).
2. *Content.Metrics* (*C.Metrics*) – If *C.VPT* is present, data from those assessments is provided as specific values, with a clear indication of the scale or metrics used (e.g., dB re 10^–6^ m/s^2^; μm,). Said scale/metric must be suitable for comparing data from other studies. In papers where the scale or metrics are not clearly stated (e.g., paper only informs that "data are presented as dB"), the paper offers sufficient information to enable readers to find it elsewhere (e.g., instrument’s brochure or leaflet, ISO standards). Additionally, data are available in either text format, graph format with labelled markers, as supplementary material, or can be provided by the authors after contact.
3. *Content.Healthy/Control* (*C.Healthy/Control*) – The paper reports on at least one study or experiment conducted with a population or group of healthy or control individuals (not daily exposed to vibrations, nor with a prior history of neuropathy). The paper reports on at least one baseline/control condition if different experimental conditions were employed. Data obtained from healthy/control individuals and/or baseline/control conditions are reported separately when applicable. In cases where data from separate groups or conditions is presented only after being combined, a justification is provided regarding said combination (e.g., the initial analysis revealed no significant differences between the results of groups/conditions).
4. *Content.Frequency* (*C.Frequency*) – The paper presents either the specific frequencies used to generate vibrations or associates each reported threshold value with its corresponding frequency. The results for each frequency are presented separately, with a clear indication of which threshold corresponds to each frequency.
5. *Content.Hand/Finger (C.Hand/Finger) –* The paper reports on at least one assessment conducted on the glabrous skin of a hand or finger and provided the results obtained from distinct locations separately. In cases where data from separate locations is presented only after being combined, a justification is provided regarding said combination (e.g., the initial analysis revealed no significant differences between the results of separate locations).

For each of the 211 records, each criterion was assessed as being either present ("Yes") or absent ("No"). All records were then organized into inclusion (Content Inclusion) and exclusion (Content Exclusion) groups. Table 2 presents all the content inclusion and exclusion groups and the number of records included in each.

**Table 2:** Content inclusion categories according to criteria and attributed number of papers

Group	C.VPT	C.Metrics	C.Healthy/Control	C.Frequency	C.Hand/Finger	No. included
Content Inclusion	Yes	Yes	Yes	Yes	Yes	39
Total included in review						39
Content Exclusion 1	Yes	No	No	No	No	8
Content Exclusion 2	Yes	No	Yes	Yes	No	25
Content Exclusion 3	Yes	No	Yes	No	No	15
Content Exclusion 4	No	No	Yes	No	No	52
Content Exclusion 5	Yes	No	Yes	Yes	Yes	30
Content Exclusion 6	No	No	No	No	No	4
Content Exclusion 7	Yes	Yes	Yes	Yes	No	3
Content Exclusion 8	Yes	Yes	No	Yes	No	1
Content Exclusion 9	Yes	No	No	Yes	Yes	2
Content Exclusion 10	Yes	Yes	Yes	No	Yes	3
Content Exclusion 11	Yes	No	Yes	No	Yes	5
Content Exclusion 12	Yes	Yes	No	Yes	Yes	5
Content Exclusion 13	No	No	Yes	Yes	Yes	7
Content Exclusion 14	No	No	Yes	No	Yes	2
Content Exclusion 15	Yes	No	No	No	Yes	1
Content Exclusion 16	Yes	Yes	Yes	No	No	1
Content Exclusion 17	Paper describes device and/or experimental protocol only, no assessments were conducted					9
Total excluded from review						173

Records in which all criteria were present were included in the Content Inclusion group. Records with at least one absent criterion were included in one of the Content Exclusion groups. These Exclusion groups were created and organized based on the different combinations of present and absent criteria found across the excluded records, resulting in 16 Content Exclusion groups. An additional exclusion group was also created to encompass papers which only described a device and/or experimental protocol, with no assessments being conducted or reported (Content Exclusion 17). Two important considerations regarding the *C.Metrics* criterion should be noted:

- Papers that presented their results in relative units, such as decibels (dB) or volts (V/Volt), without a clear reference value (e.g., 10^–6^ m/s^2^) or indication of where one can be found (e.g., procedure/instrument compliant with ISO 13091–1 recommendations), were determined to not meet the *C.Metrics* criterion. For papers where this was the only criterion not met (Content Exclusion 5 group), and such was due to this reason, if the study made use of commercial measurement devices to acquire VPT data, the authors of this review searched through publicly available sources (e.g., instrument booklets, leaflets, webpages) to find reference values for said devices. If these reference values were found, and the information matched the one provided in the paper, it was then considered to meet the *C.Metrics* criterion.
- In a similar manner, papers that did not provide concrete VPT data, either in the form of text, supplementary material, or marker labels in graphs, were determined to not meet the *C.Metrics* criterion. For papers where this was the only criterion not met (Content Exclusion 5 group), and such was due to this reason, the authors of this review contacted the corresponding authors of said papers, through the contact information indicated in those papers, to request access to the data files containing their VPT results. If the data was provided within four weeks of the initial request, and if said data files contained the required information, they were taken as part of the paper’s supplementary materials, which was then considered to meet the *C.Metrics* criterion.

**Fig. 2:**
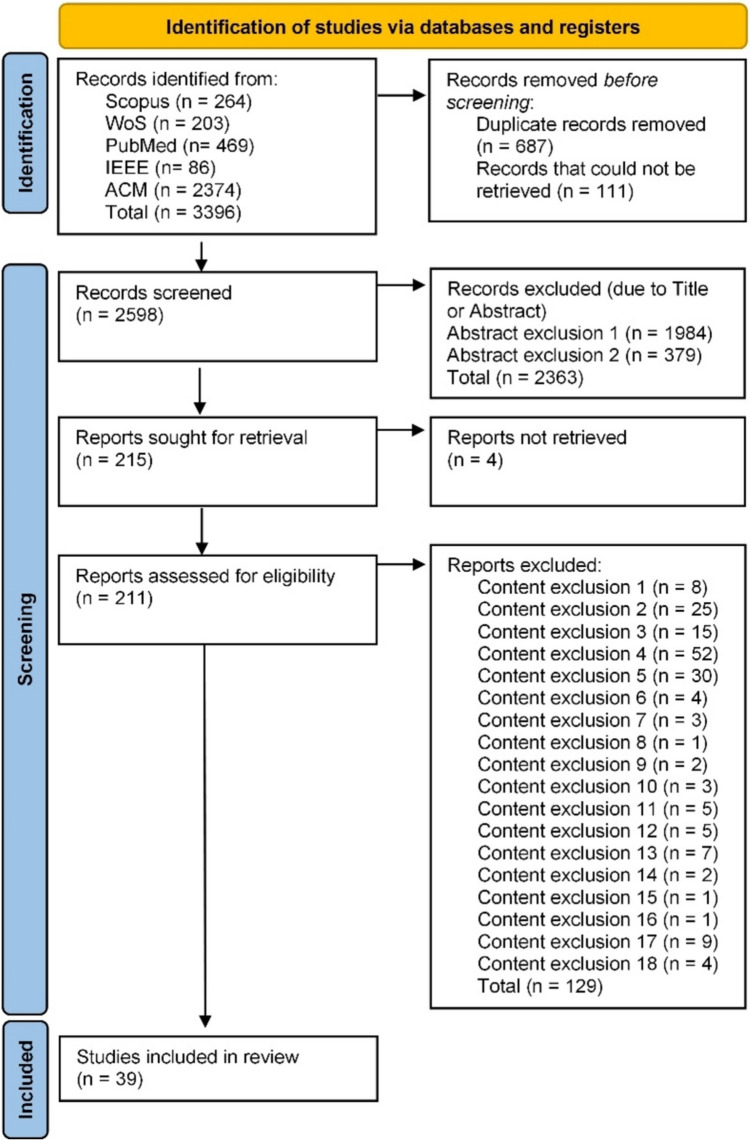
PRISMA 2020 flow diagram of the systematic review (Page et al., 2021)

From the 211 outputs, and after the content analysis, our systematic review yielded 39 final papers. The PRISMA 2020 flowchart (Page et al., 2021), presented in Fig. 2, summarizes the process, starting from the initial 3396 reports to 211, and up until the final 39 studies included in this review’s analysis.

## Content analysis

While assessing the content eligibility of all 211 retrieved reports, when possible/applicable, information regarding the following aspects was extracted: a) the goal/objective of each paper and reported studies/experiments; b) the number of healthy and/or control participants and their characteristics; and c) the characteristics of the experimental protocol(s) employed. In this section, the information extracted from the 39 studies included in this review will be presented and discussed. When dealing with papers that encompass multiple studies or experiments, we will narrow our focus only to those that match the content inclusion criteria described in “Eligibility criteria – Content” chapter of the “Methods”  Section. In such occurrences, the study, experiment, and/or experimental condition being focused on will be identified, following the same naming convention as used in its original paper.

### Study goals

Table 3 provides a summary of the stated overall goals of each of the reviewed studies. Information regarding the type of publication of each paper is also provided.

**Table 3:** Stated overall goals of each reviewed study

Reference	Publication	Goal	Group	Subgroup
Ahn et al. (2013)	Journal	Acquire normative data for the diagnosis of HAVS for the South Korean population	Human body	Characteristics
Arredondo and Perez (2017)	Journal	Assess the effect of a white-noise vibration, applied in either 1 or 4 concurrent locations, on participant’s VPTs	Experimental protocols	Other stimuli
Calder et al. (2012)	Journal	Assess sensory impairments in women with OA or RA of the hand, compared to those without hand issues, using neurophysiological and sensory perception tests, including VPT; Assess the relationship between sensory status and self-reported hand disability, strength, and dexterity	Human body	Pathologies
Chauvelin et al. (2014)	Journal	Assess the effects of technical characteristics of vibration signals in participants’ VPTs; Assess the effects of gender on VPTs	Human body	Physical responses
Clemm et al. (2019)	Journal	Assess and compare the effects of exposure to HAV on VPT results on a group of road workers with and without contact with handheld vibrating tools	Human body	Pathologies
Dahlin et al. (2015)	Journal	Assess VPT, for different frequencies, at the fingers and feet of healthy children and young adults	Human body	Characteristics
Ekman et al. (2019)	Journal	Assess the effect of conducting repeated VPT assessments, on the same fingers, over a period of 5 months, and up to 18 months, on participant’s VPT results	Experimental protocols	Procedures
Ekman et al. (2021)	Journal	Assess VPT in a healthy adult population, comparing the effects of age, sex, height, weight, footedness, handedness, and skin temperature on VPT	Human body	Characteristics
Flondell et al. (2017)	Journal	Compare VPT results from patients with different levels of CTS with results from matched healthy control participants	Human body	Pathologies
Folmli et al. (2018)	Journal	Assess the effects of tDCS, applied over the course of 5 days, on participant’s VDeT	Experimental protocols	Other stimuli
Gerhardsson et al. (2013)	Journal	Assess and compare VPT results obtained from young worked exposed to HAV tools, and healthy comparison subjects	Human body	Pathologies
Gu and Griffin (2013)	Journal	Assess the effect of probe size on VPTs for 20-Hz and 160-Hz frequencies, obtained at the sole of the foot and on the hand, and compare said VPT results between different probe sizes and locations	Experimental protocols	Procedures
Güçlü and Dinçer (2013)	Journal	Assess the effects of a masking stimulus on participant’s VPTs	Experimental protocols	Other stimuli
Haseleu et al. (2014)	Journal	Assess the effects of finger wrinkling on participants’ tactile perception by examining changes to TSA and VDeT	Human body	Physical responses
Hatzfeld et al. (2016)	Journal	Assess VPTs and JND; Assess the effects of external factors on VPTs and JND	Experimental protocols	Procedures
Held et al. (2021)	Journal	Assess and compare VPTs at the fingertips and palms of subjects with Dupuytren disease against healthy control subjects	Human body	Pathologies
Hopkins et al. (2016)	Journal	Assess VPTs for frequencies related to piano key notes at the fingertips and feet of participants with normal hearing; Compare fingertip VPT results between participants with and without hearing impairments; Assess vibrotactile perception of parts of G4 to C6 notes; Assess the possibility of presenting music through vibrotactile stimuli	Human body	Pathologies
Koszewicz et al. (2021)	Journal	Assess and compare different QST results, including VPT, obtained from the median and ulnar nerves of healthy participants	Experimental protocols	Procedures
Kowalski and Zając (2012)	Journal	Assess the effects of HAV and WBV exposure on participant’s VPTs	Experimental protocols	Other stimuli
Labbé et al. (2016)	Journal	Assess the influence of tDCS on participant’s tactile perception by examining changes to VDeT and JND	Experimental protocols	Other stimuli
Lundström et al. (2018)	Journal	Assess and compared the effects of skin thickness on VPT and TPT, obtained from a group of workers daily exposed to HAV, and a group of workers not daily exposed to HAV	Human body	Characteristics
Marcuzzi et al. (2019)	Journal	Assess and compare the test–retest reliability of using a graduated tuning fork, and an electronic vibrameter with either a hand-held or a fixed probe, to assess VDiT/VPT	Experimental protocols	Procedures
Moshourab et al. (2017)	Journal	Assess and compare different QST results, including VPT, obtained from adolescents and young adults with congenital hearing impairments, with those obtained from age-matched healthy controls	Human body	Pathologies
Niwa et al. (2021) — Experiment I	Journal	Assess the effects of delivering vibrotactile feedback to the temple on the finger’s VPTs	Experimental protocols	Other stimuli
Oh and Choi (2019)	Journal	Assess the effects of pressing force on participant’s VPTs	Experimental protocols	Procedures
Papetti et al. (2017)	Journal	Assess the effect of active pressing force on the VPT of a sine wave and a white-noise vibration	Experimental protocols	Procedures
Pra et al. (2023)	Journal	Assess the effect of contact force and vibration direction on the participant’s perception of a 250-Hz vibration	Experimental protocols	Procedures
Prsa et al. (2021)	Journal	Establish the V-shape sensitivity curves for both mice and humans; Assess the effects of changes to vibration amplitude in the perceptual responses to vibration in both mice and humans	Human body	Characteristics
Sakurai and Shinoda (2014) — ES Device Evaluation Experiment	Conference paper	Assess the effects of delivering vibrations though the edge stimulation method on VPTs	Experimental protocols	Other stimuli
Shibata (2022)	Journal	Assess the effects of HAV exposure on participant’s VPTs	Experimental protocols	Other stimuli
Shibata (2023)	Journal	Assess the effects of repeated HAV exposure on participant’s VPT	Experimental Protocols	Other stimuli
Tamrin et al. (2016)	Journal	Assess the effects of chemical exposure and contributing factors on participant’s VPT	Human body	Physical responses
Tanaka et al. (2015)	Journal	Assess the effects of presence or absence of skin-transmitted vibrations on participant’s VDeTs and JND	Experimental protocols	Other stimuli
Tanaka et al. (2016)	Conference paper	Assess the effects of providing a vibration on the forearm on participant’s VPTs at a fingertip	Experimental protocols	Other stimuli
Witte et al. (2022)	Journal	Assess the effects of different levels of HAV exposure on participant’s VPTs	Experimental protocols	Procedures
Ye and Griffin (2013)	Journal	Assess the effects of applying a 125-Hz vibration, 15-dB above threshold level, at the thenar eminence of the hand, using two different contact probe areas, on participants FBF and FST	Experimental protocols	Other stimuli
Ye and Griffin (2016)	Journal	Assess if decreases in FBF, induced by applying a 125-Hz vibration, 15 dB above threshold level, to different areas of the hand, are related to the VPT for 125-Hz vibrations at said hand areas	Human body	Physical responses
Yildiz and Güçlü (2013)	Journal	Assess the effects of different probe sizes on VPTs, measured at 3 locations of the same fingerpad	Experimental protocols	Other stimuli
Yildiz et al. (2015)	Journal	Assess the effects of active and passive movement, as well as the effect of forward masking stimulus, on participant’s VPTs	Experimental protocols	Other stimuli

*CTS *Carpal tunnel syndrome, *FBF *finger blood flow, *FST *finger skin temperature, *HAV *hand–arm vibration, *HAVS *hand–arm vibration syndrome, *JND *just-noticeable difference, *OA *osteoarthritis, *QST *quantitative sensory testing, *RA *rheumatoid arthritis, *TPT *thermotactile perception thresholds, *TSA *tactile spatial acuity, *VDeT *vibration detection threshold, *VDiT *vibration disappearance threshold, *VPT *vibration perception threshold, *WBV *whole-body vibration, *tDCS *transcranial direct current stimulation

As can be noted, the gathered studies focused on either investigating the influence of aspects of the human body and its inner workings, or the influence of aspects of the employed experimental protocols or other factors external to the human body, on VPT assessments. Regarding the former (i.e., aspects of the human), these studies can be further grouped into: (1) studies that assessed the effects of different characteristics, such as age and gender (e.g., Dahlin et al., 2015; Ekman et al., 2021); (2) studies which assessed the effect of different pathologies, such as Osteoarthritis (Calder et al., 2012), Carpal tunnel syndrome (Flondell et al., 2017), and hand–arm vibration exposure (Clemm et al., 2019); and (3) studies that assess the effects of different physical responses of the human body to certain events, such as finger wrinkling, (Haseleu et al., 2014), exposure to chemicals (Tamrin et al., 2016) and finger-blood flow level (Ye & Griffin, 2016).

As for the studies that assessed the influence of aspects of the employed experimental protocols or other factors external to the human body, these can be grouped into: (1) studies that assessed the effects of other stimuli, such as other vibrations applied either concurrently or before and/or after the test stimulus (e.g., Arredondo & Perez, 2017; Kowalski & Zając, 2012; Shibata, 2023; Ye & Griffin, 2013); and (2) studies that assessed the effects of different experimental procedures, such as different probe sizes (Gu & Griffin, 2013) or pressure/contact force (Oh & Choi, 2019; Papetti et al., 2017; Pra et al., 2023). These groups are presented in Table 3 under the Group and Subgroup columns.

As for the type of publication of each paper, all except two were published in journals, with Tanaka et al. (2016) and Sakurai and Shinoda (2014) being published in conferences.

### Participant population data

For each of the reviewed papers, information regarding the country where the study was conducted, the number and gender distribution of the participant sample, as well as age information (either in general or separated by gender group) was extracted with as much detail as possible based on the available information. These data are available in Table 1S, available as supplementary material. When applicable, the information presented in this table was further separated to distinguish between separate participant/experimental condition groups.

Data regarding the country where studies were conducted were extracted from direct indications in the text of either country, study location, ethics committee that approved the study, author’s address, or information about recruited populations. Of the 39 included studies, six (15.4%) each were conducted in Sweden and Japan, four (10.3%) were carried out in the United Kingdom (U.K.) and Germany, respectively, three (7.7%) were conducted in Turkey, two (5.1%) each were conducted in Poland, South Korea (KR), Australia, and Canada, and one study each (2.6%) was carried out in Malaysia, France, the United States of America (U.S.A.), Chile, Switzerland, Italy, and Norway. Only one paper did not provide information regarding the country where the study was conducted.

As for participant sample information, globally, across all 39 studies, data was obtained from 2301 healthy and/or control subjects, of which 1158 (50.33%) were reportedly male, 1097 (47.67%) were reportedly female, and 46 (2.00%) did not have their gender reported. Twelve of these studies were conducted with a fully male sample, while one study was comprised of only female participants. The studies with the lowest number of participants were those of Sakurai and Shinoda (2014) and Yildiz and Güçlü (2013), with seven participants each, while the study with the largest participant sample was Ekman et al. (2021)‘s study, which gathered data from 913 participants. Data from 20 subjects was gathered in four studies (Ekman et al., 2019; Haseleu et al., 2014; Shibata, 2022, 2023), three studies each collected data from ten participants (Kowalski & Zając, 2012; Oh & Choi, 2019; Yıldız et al., 2015) and eight participants (Güçlü & Dinçer, 2013; Niwa et al., 2021; Witte et al., 2022). Two studies each collected data from 120 participants (Ahn et al., 2013; Tamrin et al., 2016), 27 participants (Hatzfeld et al., 2016; Papetti et al., 2017), 19 participants (Clemm et al., 2019; Prsa et al., 2021), 13 participants (Arredondo & Perez, 2017; Labbé et al., 2016), and 12 participants (Gu & Griffin, 2013; Ye & Griffin, 2016). All other sample sizes besides these were found in one study each.

The age distribution across all 2301 subjects ranged from 8 to 90 years old, with a weighted average of 31.31 years old. As for age distribution across genders, considering only the studies that reported said information, it ranged between 18 and 75 for males (Ahn et al., 2013; Gerhardsson et al., 2013; Gu & Griffin, 2013; Güçlü & Dinçer, 2013; Held et al., 2021; Koszewicz et al., 2021; Kowalski & Zając, 2012; Lundström et al., 2018; Sakurai & Shinoda, 2014; Shibata, 2022, 2023; Tamrin et al., 2016; Witte et al., 2022; Ye & Griffin, 2016) and 22 to 76 for females (Calder et al., 2012; Güçlü & Dinçer, 2013; Held et al., 2021; Koszewicz et al., 2021; Niwa et al., 2021), with a weighted average of 24.91 and 55.07 years old, respectively.

### Assessment instruments, temperatures, pressure/contact force, and skin indentation

Table 4 presents an overview regarding the reported: instrumentation used to measure VPT; probe size (diameter, in mm, converted when required); skin temperature at the contact location (°C); room temperature (°C); pressure applied between the contact area and the probe (either as N, N/cm^2^, mN, or grams); and the skin indentation of the probe on the skin (in mm, converted when required). Information about the instrument’s name, model, and maker was provided as found in each respective paper. Studies where the assessment instrument was created by the study’s authors or their teams were labeled as custom-made apparatuses (for more information on these instruments, please see their respective papers). Additionally, for studies that used non-electronic equipment (e.g., tuning forks) to assess VPT, those instruments were not reported due to their measurement process and units.

**Table 4:** Assessment instruments and temperatures

Reference	Device(s) used for assessments	Probe diameter	Use of surround/aperture (diameter), temperature, area pressure/force (either N, N/cm^2^, mN, or grams), skin indentation (mm)
Ahn et al. (2013)	Mini-shaker Type 4810 vibration generator (Brüel and Kjær, Denmark)	2.6 mm	No; 24–28 °C room temperature; 1.5-mm skin indentation
Arredondo and Perez (2017) — Experiment 1	Custom-made apparatus	1.5 mm	Yes; 200 mN
Calder et al. (2012)	Jtech vibrometer (Vibrometer PCV50; Jtech, Salt Lake City, UT)	1 mm	Yes (2 mm); 23 ± 2 °C room temperature
Chauvelin et al. (2014)	Custom-made apparatus	—	—
Clemm et al. (2019)	VibroSense Meter® (VibroSense Dynamics, Malmö, Sweden)	3 mm	20–30 °C room temperature (following ISO 13091–1); 1.5 ± 0.8 mm skin indentation (following ISO 13091–1)
Dahlin et al. (2015)	VibroSense Meter® (Vibrosense Dynamics, Malmö, Sweden)	4 mm	27–35 °C skin temperature; 20–22 °C room temperature; 0.15 ± 0.09 N, 1.5-mm skin indentation
Ekman et al. (2019)	VibroSense Meter® (Vibrosense Dynamics, Malmö, Sweden)	4 mm	20–22 °C room temperature; 0.15 ± 0.09 N, 1.5-mm skin indentation
Ekman et al. (2021)	VibroSense Meter® I (VibroSense Dynamics, Malmö, Sweden)	4 mm	No surround; 20–22 °C skin temperature; 0.15 ± 0.09 N, 1.5-mm skin indentation
Flondell et al. (2017)	VibroSense Meter® (Vibrosense Dynamics, Malmö, Sweden)	—	≥ 30 °C skin temperature; 0.15 ± 0.09 N, 1.5-mm skin indentation
Folmli et al. (2018)	Custom-made apparatus	6 mm	Yes (8 mm); 21 °C room temperature
Gerhardsson et al. (2013)	HVLab Tactile Vibrometer, UK	4 mm	Yes; > 28 °C skin temperature; 1 N
Gu and Griffin (2013)	HVLab Vibrotactile Perception Meter (VPM, University of Southampton)	1 mm; 3 mm; 6 mm; 10 mm	Yes; 25.8–30.9 °C skin temperature; 24–26 °C room temperature; 2 N
Güçlü and Dinçer (2013)	Custom-made apparatus	4 mm	No; 32–36 °C skin temperature; 0.5 mm skin indentation
Haseleu et al. (2014)	Custom-made apparatus	10 mm	30 g applied to the skin
Hatzfeld et al. (2016)	Custom-made apparatus	19.22 mm	Yes (1 mm); 22 °C surrounding air temperature; 1 N
Held et al. (2021)	Custom-made apparatus	8.9 mm	No; Varied room temperature; 0.5 N
Hopkins et al. (2016) — Experiment I (fingertip)	Custom-made apparatus	20 mm	
C1 to G2 notes			Yes; 20–36 °C skin temperature
C3 to C6 notes			Yes; 24–36 °C skin temperature
Koszewicz et al. (2021)	VSA–3000 Vibratory Sensory Analyzer (Medoc, Israel)	≈12.46 mm	≥ 32 °C skin temperature; 21–23 °C room temperature
Kowalski and Zając (2012)	—	—	27–35 °C skin temperature; 20–30 °C room temperature; 0.1 N
Labbé et al. (2016) — Vibrotactile Detection Task	Custom-made apparatus	3.1 mm	Yes (20 × 20 mm); 0.5-mm skin indentation
Lundström et al. (2018)	Custom-made apparatus	6 mm	No; ≥ 28 °C skin temperature; 3.5 N/cm^2^
Marcuzzi et al. (2019)	Vibrameter (Model VSA3000, Medoc, Yamai Ramat, Israel)	—	23 ± 1 °C room temperature
Moshourab et al. (2017)	Custom-made apparatus	8.21 mm	20–30 °C room temperature; 30 g applied to the skin
Niwa et al. (2021) — Experiment I	Custom-made apparatus	—	No; 0.5 N
Oh and Choi (2019)	Custom-made apparatus	13.59 mm	No; 0.2 N, 2 N, 4.9 N, 7.8 N, 10.8 N
Papetti et al. (2017)	Custom-made apparatus	—	1.9 N, 8 N, 15 N
Pra et al. (2023)	Custom-made apparatus	—	0.5 N, 4.9 N
Prsa et al. (2021) — Detection Task in Humans	Custom-made apparatus	3 mm	—
Sakurai and Shinoda (2014) — ES Device Evaluation Experiment	Custom-made apparatus	"15-mm square flat plane"	No; 1 N
Shibata (2022)	Rion Type AU-06 (Rion Inc., Tokyo, Japan)	4 mm	Yes (1.5 ± 0.6 mm); 23 ± 2 °C room temperature; 0.5 ± 0.3 N
Shibata (2023)	Rion Type AU-06 (Rion Inc., Tokyo, Japan)	4 mm	Yes (1.5 ± 0.6 mm); 23 ± 2 °C room temperature
Tamrin et al. (2016)	HVLab Tactile Vibrometer, UK*	—	20–30 °C skin temperature; 0.1 N
Tanaka et al. (2015) — Detection Experiment	Multilayer piezoelectric actuators (PZA12-1T, Matsusada Precision Inc.)	5 mm	No; 0.5 N
Tanaka et al. (2016)	Custom-made apparatus	—	0.34 N
Witte et al. (2022)	VibroSense Meter® II (VibroSense Dynamics, Malmö, Sweden)	—	25.6 ± 0.9 °C room temperature; 1.5-mm indentation
Ye and Griffin (2013)	HVLab Vibrotactile Perception Meter (VPM, University of Southampton)	3 mm; 6 mm	Yes (26 mm); > 30 °C skin temperature; 25.1 ± 0.4 °C room temperature; 5 N
Ye and Griffin (2016)	HVLab Vibrotactile Perception Meter (VPM, University of Southampton)	6 mm	Yes (2 mm); 25.2 ± 0.5 °C room temperature; 2 N
Yildiz and Güçlü (2013)	Custom-made apparatus	2 mm; 4 mm; 7 mm	30–34 °C skin temperature; 0.5-mm skin indentation
Yildiz et al. (2015)	Custom-made apparatus	14 mm	Yes (2 mm); 33.1 ± 0.8 °C skin temperature

— Not mentioned or specified, * this device is an HVLaB Vibrotactile Perception Meter (VPM, University of Southampton), but this was nomenclature used in the study, ≈ Diameter calculated from provided probe area

#### Assessment instruments

Apparatuses classified as custom-made were used in 20 of the 39 reviewed studies (51.28%), while one study (2.56%) did not report information regarding which instrument was used to measure VPTs. The remaining 18 studies (46.15%) made use of commercially available devices (for brevity, separate versions, and/or models of the same branded devices were taken as being the same), most predominantly of which were the VibroSense Meter ® devices. VibroSense Meter ® devices were employed in six studies (15.38% of 39) and HVLaB (Vibro)meters were used in five studies (12.82% of 39). The Rion Type AU-06 and the VSA–3000 Vibratory Sensory Analyzer were each used in two studies (5.13% of 39 each). All other commercially available instruments—Jtech vibrometer, Mini-shaker Type 4810 vibration generator, and Multilayer piezoelectric actuators—presented in Table 4 were utilized in one study each.

#### Probe size and surround aperture

Probe size refers to the size of the probe head/tip in contact with the subject’s skin, through which vibrations are transmitted. When circular probes, the most common type of probe shape, are used, their size is often reported as either the diameter or radius of the circular surface. For simplicity, the probe size of circular probes reported here will refer to their diameter unless otherwise stated. Circular probes, across the 39 reviewed papers, were employed in 38 of them, with their size varying between 1 and 20 mm. Ten studies did not report the probe size used in their assessments. The most often used probe size across the 39 reviewed studies was 4 mm (used in eight studies), followed by 6 mm (used in five studies), 3 mm (used in four studies), and 10 mm and 1 mm (used in two studies each). All other probe sizes—20, 19.22, 14, 13.59, 12.46, 8.9, 8.21, 7, 3.1, 2.6, 2, and 1.5 mm—were used in only one study each. Additionally, Sakurai and Shinoda (2014)‘s ES Device Evaluation Experiment used a 15 × 15-mm squared probe.

To control the skin indentation, as well as the static force exerted between the probe and the skin, a rigid surround can be employed around the probe head, which requires an aperture through which the probe can protrude. Regarding the use of a surround, this information was reported in 22 of the 39 papers, with 13 indicating the use of a surround and nine mentioning that a surround or aperture was not used. On the papers that used surround, four did not provide information about its diameter, three reported it was 2 mm, and two reported it to be 1.5 mm. The remaining diameter sizes—1, 8, 26, and 20 × 20 mm—were all reported in one study each.

#### Skin and room temperature

Considering the reported skin temperature during VPT assessments, 25 studies did not provide this information. Of the studies that did, in turn, five reported it as being greater/less than or equal to a temperature value, one study reported the average temperature measured, and nine reported it as a range of the minimum–maximum temperatures registered. Across these nine studies, the lowest minimum temperature reported was 20 °C, in three studies, while the maximum was 36 °C, in three studies as well.

The room temperature, in turn, was mostly reported as either the average temperature, alongside its standard deviation (nine studies) or as a range of the minimum–maximum temperatures registered (eight studies). Twenty-two studies did not report this information. Of the nine studies that reported average temperature, the most common was 23 °C, in four studies, with all others being reported in one study each, the lowest being 21 °C, and the highest 25.6 °C. As for the eight studies that reported a temperature range, the lowest minimum temperature reported was of 20 °C, in five studies, and the maximum was 30 °C, in three studies.

#### Pressure/contact force and skin indentation

In terms of the reported pressure or force applied to the skin at the contact point, 15 studies did not report this information, and four studies reported units of measurement other than Newtons (N), which was the most reported unit (reported in 20 studies). Other units were grams, used in two studies, milli-newton (mN) and N/cm^2^, used in one study each. In studies that did report average pressure or force using Newtons (N), it ranged from 0.1 to 15 N, with a pressure or force of 0.5 N being reported in five studies, 0.15 N being reported in four studies, 2 N and 1 N being reported in three studies each, and 4.9 N and 0.1 N being reported in two studies each. All other pressures or forces—0.2, 0.34, 1.9, 5, 7.8, 8, 10.8, and 15 N—reported in Table 4 were used once each.

As for the skin indentation (i.e., the temporary compression or deformation of the skin) caused by the probe head/tip, when reported, was either 1.5 mm (seven studies) or 0.5 mm (three studies), with the remaining 29 studies not reporting this information.

### Experimental methodology

Table 5 summarizes information regarding the experimental methodology of each of the reviewed studies, namely: the experimental method or (psychophysical) algorithm that controlled how assessments were performed; in which units were the obtained VPT results expressed; at what intensity was the stimuli at on the first trial of each assessment; and the length of the step sizes or the change rates between trials of each assessment.

**Table 5:** Experimental methodology

Reference	Experimental method/algorithm	Results expressed in	Stimuli starting intensity	Step size/Change rate
Ahn et al. (2013)	Von Békésy	dB (re. 10^–6^ m/s^2^, per ISO 13091–1)	Below expected threshold (N/S)	3 dB/s
Arredondo and Perez (2017)—Experiment 1	2AFC (Staircase paradigm, 1 Down/1 Up rule)	µm	—	—
Calder et al. (2012)	Staircase algorithm (2AFC paradigm, 1 Down/1 Up rule)	µm	Below expected threshold (N/S)	—
Chauvelin et al. (2014)	Staircase method (rule not mentioned)—VPT assessed by varying frequency instead of amplitude	Hz	500 Hz	Half of not yet tested frequency range, according to the prior test Final step size: 4 Hz
Clemm et al. (2019)	Von Békésy	dB (re. 10^−6^ m/s^2^)	—	3 dB/s
Dahlin et al. (2015)	Von Békésy	dB (re. 10^−6^ m/s^2^)	80 dB	3 dB/s
Ekman et al. (2019)	Von Békésy	dB (re. 10^–6^ m/s^2^, per ISO 13091–1)	80 dB	3 dB/s
Ekman et al. (2021)	Von Békésy	dB (re. 10^−6^ m/s^2^)	100 dB	3 dB/s
Flondell et al. (2017)	Von Békésy	dB (re. 10^–6^ m/s^2^, per VibroSense’s parameters)	80 dB	3 dB/s
Folmli et al. (2018)	Method of limits	µm	Above expected threshold (N/S)	For 200 Hz – > 0.51 µm/s For 30 Hz – > 3.15 µm/s
Gerhardsson et al. (2013)	Von Békésy	m/s^2^	Below expected threshold (N/S)	3 dB/s
Gu and Griffin (2013)	Von Békésy	ms﻿^−2^ r.m.s	Below expected threshold (N/S)	≤ 1st rev. – > 5 dB/s > 1st rev. – > 3 dB/s
Güçlü and Dinçer (2013)	2AFC (staircase paradigm, 3 Down/1 Up rule, not necessarily consecutive)	dB (re. 1 µm peak)	—	1 dB
Haseleu et al. (2014)	2IFC	µm	16.158 µm	Decrease—factor of 0.79 Increase—factor of 1.26
Hatzfeld et al. (2016)	Staircase method (3IFC paradigm, 2 Down/1 Up rule)	1 mN	15 dB above reference stimulus	≤ 4th rev. – > 3 dB > 4th rev. – > 1 dB
Held et al. (2021)	Psi-marginal adaptative algorithm (2AFC paradigm, 1 Up/ 1 Down rule)	µm	Above expected threshold (N/S)	—
Hopkins et al. (2016) — Experiment I (fingertip)	Staircase method (2AFC paradigm, 1 Down/1 Up rule)	dB (re. 10^−6^ m/s^2^)	Familiarization stage—Above expected threshold level (N/S) Stage 1—10dBV below lowest detection level on Familiarization stage Stage 2—10dBV below lowest detection level on Stage 1	Familiarization stage ≤ 1st rev. – > 20 dBV 1st < X ≤ 2nd rev. – > 2 dBV Stages 1 & 2: 2 dBV
Koszewicz et al. (2021)	Method of limits	µm	0 dB	0.3 μm
Kowalski and Zając (2012)	Von Békésy	dB (re. 10^−6^ m/s^2^)	—	—
Labbé et al. (2016) – Vibrotactile Detection Task	Staircase algorithm (2AFC paradigm, Up/Down rule not specified)	µm	N/A	N/A
Lundström et al. (2018)	Von Békésy	dB (re. 10^−6^ m/s^2^)	Below expected threshold (N/S)	3 dB/s
Marcuzzi et al. (2019)	Method of Limits	µm	0 µm	0.3 µm/s
Moshourab et al. (2017)	2AFC (Staircase paradigm, 1 Down/1 Up rule)	µm; nm	10 Hz – > 7.18 µm 125 Hz – > 2.84 µm	—
Niwa et al. (2021) — Experiment I	Staircase method (2AFC paradigm, 1 Down/1 Up rule)	µm peak-to-peak	Downward trials: 8.8 µm Upward trials: 1.4 µm	0.2 µm
Oh and Choi (2019)	Staircase method (2AFC paradigm, 2 Down/1 Up rule)	dB (re. 1.0 µm)	Above expected threshold (4 × above)	≤ 3rd rev. – > 4 dB > 3rd rev. – > 1 dB
Papetti et al. (2017)	Staircase method (2AFC paradigm, 2 Down/1 Up rule)	dB RMS (re. 10^−6^ m/s^2^)	Above expected threshold (N/S)	2 dB
Pra et al. (2023)	Binary choice (detected/not detected)	dB RMS (re. 10^−6^ m/s^2^)	N/A	N/A
Prsa et al. (2021) — Detection Task in Humans	2AFC (Staircase paradigm, 3 Down/1 Up rule)	µm	Maximum amplitude of tested frequency	≤ 1st rev. – > 12 dB > 1st rev. – > previous amplitude/2 Minimum step size – > 3 dB
Sakurai and Shinoda (2014) — ES Device Evaluation Experiment	Method of limits	µm	1st: 0V 2nd: above expected threshold level (N/S)	2V
Shibata (2022)	Von Békésy	dB (re. 10^–6^ m/s^2^, per Rion AU-06’s parameters)	Below expected threshold (N/S)	2.5 dB/s
Shibata (2023)	Von Békésy	dB (re. 10^–6^ m/s^2^, per Rion AU-06’s parameters)	Below expected threshold (N/S)	2.5 dB/s
Tamrin et al. (2016)	Von Békésy	m/s^2^	Below expected threshold (N/S)	—
Tanaka et al. (2015) – Detection Experiment	Method of limits	µm peak-to-peak	Above expected threshold runs (0.130 µm peak to peak) Below expected threshold runs (0.026 µm peak to peak)	0.001 µm/s
Tanaka et al. (2016) — Session 3	Method of limits	µm	Above expected threshold, in descending trials Bellow expected threshold, in ascending trials	—
Witte et al. (2022)	Von Békésy	dB (re. 10^–6^ m/s^2^, per VibroSense’s parameters)	—	—
Ye and Griffin (2013)	Von Békésy	ms﻿^−2^ r.m.s	—	3 dB/s
Ye and Griffin (2016)	Von Békésy	ms﻿−2 r.m.s	Above expected threshold (N/S)	3 dB/s
Yildiz and Güçlü (2013)	2IFC (Staircase paradigm, 3 Down/1 Up rule, not necessarily consecutive)	dB (re. 1 µm peak)	—	1 dB
Yildiz et al. (2015)	2IFC (Staircase paradigm, 3 Down/1 Up rule, not necessarily consecutive)	µm	Above expected threshold (N/S)	≤ 1st rev. – > 5 dB > 1st rev. – > 1 dB
*AFC *Answer/alternative forced-choice task, *IFC *interval forced-choice task, *N/S *not specified, *N/A *not applicable, — not mentioned or specified, *rev. *reversal, *re. *relative to				

#### Experimental methods used to determine VPT

The experimental psychophysical methods extracted for each study were based on both the order in which the authors of the respective papers first referred to them and on interpretations regarding said methodologies from the authors of this review. For example, Niwa et al. (2021) reportedly employed the Staircase method to assess VPTs throughout their Experiment I, with participants having to answer, on every trial, if a vibration was perceptible or not. Thus, their methodology was primarily classified as being the Staircase method, and since the “yes or no” question style can be interpreted as a form of a two alternative forced choice (2AFC) task, although not explicitly mentioned by the authors, the 2AFC task was considered as a paradigm for this study. Papetti et al. (2017), in turn, also reported using the Staircase method to assess thresholds but specified that responses were provided following a 2AFC task. As the Staircase method was the first one to be identified in their manuscript, their methodology was classified as being the Staircase method following a 2AFC paradigm as well. In a similar vein, Moshourab et al. (2017) reportedly employed a 2AFC task to assess VPTs, following a staircase paradigm to control the amplitude of the following trials. Thus, their methodology was primarily classified as being the 2AFC task, following a staircase paradigm.

However, it should be noted that the descriptions attributed to a 2AFC task provided by both Papetti et al. (2017) and Moshourab et al. (2017), among other authors, are similar to descriptions of a two interval forced choice (2IFC) task provided by other authors, such as Yildiz and Güçlü (2013), who reportedly employed a 2IFC task—i.e., a vibration stimulus was presented to participants during one of two intervals, with subjects having to respond which interval contained a vibration—to assess VPTs. Due to the discrepancies found between the identification of some methodologies and their subsequent description over the same paper, which are likely a result of the similarities found across both methodologies and how said methods could be interpreted or referred to, during the analysis of this review, AFC tasks and IFC tasks will both be considered under the umbrella of AFC tasks, for simplicity. However, these two methods will be presented as distinct in Table 5 for transparency. Additionally, in a similar manner, for the remainder of this review, methodologies presented as following an “up and down” rule, algorithm, or methodology, will be interpreted as employing either the Staircase method, if such is the main methodology of the study, or as following a Staircase paradigm, if using such rules to dictate the stimuli amplitude of trials following another methodology considered as main, such as a 2AFC task. This interpretation is reflected in Table 5 and the following analyses.

Throughout the 39 reviewed papers, the von Békésy algorithm was used to assess VPT in 16 studies (41.03% of papers), almost half of the reviewed papers. The second most often used method was the staircase method (different “up and down” rules were all counted as "Staircase method"), employed in eight studies (20.51%), followed by the AFC method (different amounts of alternatives or intervals were all counted as “AFC method”), reported in seven studies (17.95%), and the method of limits, used in six studies (15.38%). Two other methods were employed in one study each, namely the Binary choice method and the Psi-marginal adaptative algorithm.

#### Units of report

While distinguishing if VPT results are reported as either peak, peak-to-peak, or root-mean-squared measures is important for the accurate conversion and comparison of assessment results, for this review, papers that did not report these distinctions were nevertheless considered for the following analyses. For more information regarding the importance of these distinctions, and how to properly convert results between them, please consult Griffin (1990).

The two most common units of measurement used to report VPT results were (1) microns (µm), used in 15 studies; and (2) decibels (dB) relative to 10^–6^ m/s^2^, used in 14 studies. It should be noted, however, that not all studies that reported data in dB relative to 10^–6^ m/s^2^ did so explicitly. In some cases, this unit of report was inferred by either checking the information available regarding the instrumentation used in the study—such was the case in Flondell et al. (2017), Shibata (2022, 2023), and Witte et al. (2022)—or assuming so when studies mentioned following the ISO 13091–1 guidelines—as in Ahn et al. (2013) and Ekman et al. (2019). Other units of measurement used in more than one study were m/s^2^, used in five studies (m/s^2^ and ms^−2^ taken as equal, for brevity), or dB relative to 1.0 µm, used in three studies. Seventeen studies, in total, reported their VPT results as dB, alongside its reference value.

Regarding the approaches towards starting amplitude values (i.e., what is the amplitude of the initial stimulus subjects are exposed to), these were specifically reported by 10 of the 39 studies, using the same unit of measurement as the VPT results, and without mention if values were above or below expected threshold levels. On the other hand, seven studies did not provide any information regarding initial stimuli amplitude. In two other studies, namely Labbé et al. (2016) and Pra et al. (2023), the stimuli amplitude of the first trial was randomized due to the study’s methodological approach. Ten studies reported the initial stimuli amplitude simply as starting from above the expected threshold level, with no specific value, while nine studies reported it as starting from below the expected threshold. In Tanaka et al.’s (2015) Detection Experiment, subjects were exposed to starting amplitudes above and below expected threshold levels, in different conditions, with the values for these initial levels being reported. This approach was also used in Tanaka et al. (2016)‘s Session 3, albeit, in this study, the specific values for initial stimulus levels were not reported. Additionally, in Sakurai and Shinoda (2014)‘s ES Device Evaluation Experiment, for all conditions, the authors reported that the first measurements began from an amplitude of 0 V—a different unit from the unit used to report assessment results—while the second measurements were conducted in a decreasing manner, starting from a well-defined amplitude.

#### Step sizes/change rates

To control the changes between amplitude increases and decreases, in response to participants' responses, a common approach is to use pre-defined step sizes or change rates. Step sizes are employed in procedures like the Staircase algorithm, which uses intermittent stimulation, with "steps" referring to the distance between the current stimuli amplitude and the amplitude of the following trial. Change rates, in turn, are used in procedures like the von Békésy algorithm, which employs constant stimuli, with the change rate referring to how quickly the stimuli amplitude varies during a condition and how large those changes are. In some cases, researchers might also opt to use two different values of step sizes or change rates, an initial larger value, to let participants reach the values around the threshold level quicker, and a second (or even third) smaller value (henceforth referred to as follow-up step size or change rate, respectively), triggered after a set number of reversals (i.e., when the stimulus changes from increasing to decreasing, or vice versa), which is meant to zone in on the threshold level with more precision (Morioka & Griffin, 2002). After a condition is finished, the following condition starts once again by using the initial step sizes or change rates, restarting the whole process once again.

Regarding the step sizes or the change rates employed in the reviewed studies, eight of the 39 studies did not report this information. In two papers, Labbé et al. (2016) and Pra et al. (2023), the use of either step sizes or change rates was not applicable due to the employed methodology.

On the other hand, seven studies used both initial and follow-up step sizes or change rates, with this change occurring most often after the first reversal (four studies). In these studies, while all reported initial step sizes or change rates were used equally once each—3 dB, 4 dB, 5 dB, 12 dB, 20 dBV, 5 dB/s, and "half of the not yet tested frequency range, according to the previous test" (Chauvelin et al., 2014, p. 874), the same could not be said for the follow-up step size or change rate, with 1 dB being the value most often used (three studies). The other follow-up values were used equally once each, those being 2 dBV, 3 dB, and 3 dB/s, and the previous amplitude/2.

Nineteen studies reported that a singular step size or change rate was constantly used throughout all assessments/conditions (i.e., no follow-ups were employed). Of these 19 studies, the most often used step size was 1 dB (two studies), while the most often used change rate was 3 dB/s (ten studies). Lastly, in a singular case, Haseleu et al. (2014) reportedly made use of a singular step size for increases in stimuli amplitude, as well as of a singular step size with a different magnitude for decreases in stimuli amplitude as well.

### Hand locations

Table 6 provides information regarding which papers conducted assessments on the little (D5), ring (D4), middle (D3), index (D2), thumb (D1), thenar eminence (T.E.), and distal palmar flexion crease (DPFC.) of the participant’s left and/or right hands. Similarly, Table 7 provides information regarding which papers conducted assessments on the D5 to D1 digits of the participant’s non-dominant and/or dominant hands (the T.E. and DPFC. were not assessed on these hands in any of the reviewed studies).

**Table 6:** Locations on the left/right hand where VPTs were assessed, per paper

Reference	Overall total	Left hand								Right hand							
		D5	D4	D3	D2	D1	T.E	DPFC	Total	D5	D4	D3	D2	D1	T.E	DPFC	Total
Arredondo and Perez (2017) — Experiment 1	1												P.				1
Dahlin et al. (2015)	2									P.			P.				2
Ekman et al. (2019)	2									P.			P.				2
Ekman et al. (2021)	3						✓		1	P.			P.				2
Gerhardsson et al. (2013)	4	P.			P.				2	P.			P.				2
Gu and Griffin (2013)	1						✓		1								
Güçlü and Dinçer (2013)	1			P.					1								
Haseleu et al. (2014)	1												✓				1
Held et al. (2021)	7		P.	P.				✓	3		P.	P.	P.			✓	4
Koszewicz et al. (2021)	4	P.			P.				2	P.			P.				2
Kowalski and Zając (2012)	1												P.				1
Labbé et al. (2016) — Vibrotactile Detection Task	1			Center of DP.					1								
Lundström et al. (2018)	1												P.				1
Marcuzzi et al. (2019)	2				DIJ.; P.				1				DIJ.; P.				1
Prsa et al. (2021) — Detection Task in Humans	1												P.				1
Sakurai and Shinoda (2014) — ES Device Evaluation Experiment	1												P.				1
Shibata (2022)	1												P.				1
Shibata (2023)	1												P.				1
Witte et al. (2022)	2									P.			P.				2
Ye and Griffin (2013)	1														✓		1
Ye and Griffin (2016)	2												P.		✓		2
Yildiz and Güçlü (2013)	1			Tip of DP.; Twirl of DP.; Lower end of DP.					1								
**Total**	41	2	1	4	3		2	1	13	6	1	1	17		2	1	28

*P. *pulp, *DP. *distal phalanx, ✓ location mentioned, but area not specified, *D5 *little finger, *D4 *ring finger, *D3 *middle finger, *D2 *index finger, *D1 *thumb, *T.E. *thenar eminence, *DPFC. *distal palmar flexion crease, *DIJ. *distal interphalangeal joint

**Table 7:** Locations on the non-dominant/dominant hand where VPTs were assessed, per paper

Reference	Overall total	Non-dominant hand						Dominant hand					
		D5	D4	D3	D2	D1	Total	D5	D4	D3	D2	D1	Total
Ahn et al. (2013)	4	P.			P.		2	P.			P.		2
Calder et al. (2012)	2							DP.			DP.		2
Chauvelin et al. (2014)	1										P.		1
Clemm et al. (2019)	4	P.			P.		2	P.			P.		2
Flondell et al. (2017)	2							P.			P.		2
Folmli et al. (2018)	2			P.			1			P.			1
Hatzfeld et al. (2016)	1										P.		1
Hopkins et al. (2016) — Experiment I (fingertip)	1									P.			1
Moshourab et al. (2017)	1							P.					1
Niwa et al. (2021) — Experiment I	1										P.		1
Oh and Choi (2019)	1										P.		1
Papetti et al. (2017)	1										P.		1
Pra et al. (2023)	1										Tip		1
Tamrin et al. (2016)	1								DP.				1
Tanaka et al. (2015) — Detection Experiment	1										P		1
Tanaka et al. (2016) — Session 3	1										P		1
Yildiz et al. (2015)	1			DP.			1						
Total	26	2		2	2		6	5	1	2	12		20

*P.* Pulp, *DP.* distal phalanx, *D5* little finger, *D4* ring finger, *D3* middle finger, *D2* index finger, *D1* thumb

This distinction between right, left, dominant, and non-dominant hand is made following the same information provided in each paper. When mentioned, a more exact description of where the probe was placed in each location was also included. It should be noted that, in their study, Gu and Griffin (2013) state that assessments were conducted at two locations on the left hand, namely the thenar eminence and at a fingertip. However, the authors do not provide concrete information regarding which precise fingertip was studied. Thus, this review will only consider the information provided by the authors regarding the thenar eminence to avoid making wrongful assumptions.

Most studies (64.10%, i.e., 25 of 39) focused on conducting assessments in only one location of any hand. Five studies (12.82% of 39) carried out assessments on locations of both left and right hands, and three studies (7.69% of 39) conducted assessments on both dominant and non-dominant hands. Two studies in particular, Marcuzzi et al. (2019) and Yildiz and Güçlü (2013) carried out assessments on 2 and 3 positions at the same hand location, respectively (see Table 6).

**Fig. 3:**
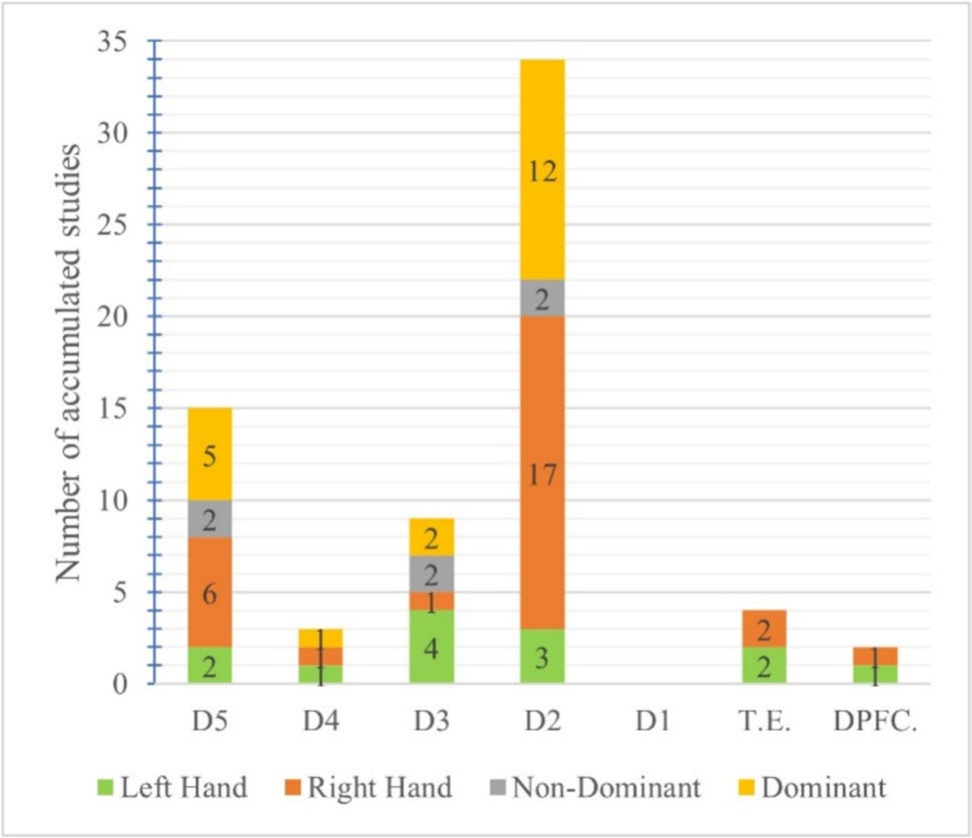
Number of studies assessing VPTs at each hand location (D5 = little finger; D4 = ring finger; D3 = middle finger; D2 = index finger; D1 = thumb; T.E. = thenar eminence; DPFC. = distal palmar flexion crease)

Of the 39 reviewed studies, 22 of them (56.41%) carried out assessments on at least one location of the left and/or the right hand. Of these 22, four only assessed locations on the left hand, 13 only assessed locations on the right hand, and five assessed locations on both hands. Of these 22 studies, as can be seen in Fig. 3, the index finger is the hand location most often used for VPT assessments (left hand: three studies; right hand: 17 studies), followed by the little finger (left hand: two studies; right hand: six studies), and the middle finger (left hand: five studies; right hand: two studies). A similar pattern is found in papers that assessed participants' non-dominant and/or dominant hands.

Of the 39 reviewed studies, 17 (43.59% of 38) assessed at least one location of the non-dominant and/or dominant hands. Of these 17, one study only assessed locations on the non-dominant hand, 13 studies only assessed locations on the dominant hand, and three studies assessed locations on both the non-dominant and dominant hands. Throughout these 17 studies, as can be seen in Fig. 3, the index finger was also the one analyzed the most often (non-dominant hand: two studies; dominant hand: 12 studies), followed by the little finger (non-dominant hand: two studies; dominant hand: five studies) and the middle finger (dominant hand: two studies; non-dominant hand: two studies). Figure 3 presents a chart summarizing this information, organized by Hand Location.

Lastly, no VPT assessments were reportedly carried out at the Right or Left Hand’s D1, at the Non-Dominant Hand’s D4, D1, T.E., and DPFC., nor at the Dominant Hand’s D1, T.E., and DPFC.

### Assessed frequencies

Tables 8 and 9 contain information regarding at which frequencies VPTs were assessed in each of the reviewed papers. Table 8 contains frequencies between 4 Hz (the smallest frequency reported) and 250 Hz, while Table 9 contains frequencies between 261.6 Hz and 1046.5 Hz (the highest frequency reported). It should be noted that, due to their methodological approach of manipulating vibration frequency instead of vibration amplitude, Chauvelin et al. (2014) reportedly studied a range of frequencies ranging from 1 to 1000 Hz. While an interesting approach to assessing VPTs, as specific frequencies were not constantly studied, this review will not take into consideration Chauvelin et al. (2014)‘s study when describing the studies’ frequencies. Thus, only 38 studies will be considered in the following sections.

**Table 8:** Frequencies (≤ 250 Hz) at which VPT (or similar) were assessed

Reference	Total	Frequency (Hz)																												
		4	5	8	10	16	20	25	30	31.5	32	32.5	32.7	40	49	50	64	65.4	75	80	98	100	120	125	130.8	150	160	196	200	250
Ahn et al. (2013)	3									x														x						x
Arredondo and Perez (2017) — Experiment 1	1								x																					
Calder et al. (2012)	1															x														
Chauvelin et al. (2014)^1^																														
Clemm et al. (2019)	7			x		x					x						x							x						x
Dahlin et al. (2015)	7			x		x						x					x							x						x
Ekman et al. (2019)	7			x		x					x						x							x						x
Ekman et al. (2021)	7			x		x					x						x							x						x
Flondell et al. (2017)	7			x		x					x						x							x						x
Folmli et al. (2018)	2								x																				x	
Gerhardsson et al. (2013)	2									x														x						
Gu and Griffin (2013)	2						x																				x			
Güçlü and Dinçer (2013)	2													x																x
Haseleu et al. (2014)	2				x																			x						
Hatzfeld et al. (2016)	9		x		x		x		x											x							x			
Held et al. (2021)	2																													x
Hopkins et al. (2016) — Experiment I (fingertip)	11												x		x			x			x				x			x		
Koszewicz et al. (2021)	1																					x								
Kowalski and Zając (2012)	5	x					x			x												x		x						
Labbé et al. (2016)—Vibrotactile Detection Task	1						x																							
Lundström et al. (2018)	7			x		x					x						x							x						x
Marcuzzi et al. (2019)	1																						x							
Moshourab et al. (2017)	2				x																			x						
Niwa et al. (2021) — Experiment I	1																									x				
Oh and Choi (2019)	2													x																x
Papetti et al. (2017)	1																													x
Pra et al. (2023)	1																													x
Prsa et al. (2021) — Detection Task in Humans	14				x			x								x			x			x							x	
Sakurai and Shinoda (2014) — ES Device Evaluation Experiment	1								x																					
Shibata (2022)	2									x														x						
Shibata (2023)	2									x														x						
Tamrin et al. (2016)	2									x														x						
Tanaka et al. (2015) — Detection Experiment	1																													x
Tanaka et al. (2016) — Session 3	1															x														
Witte et al. (2022)	5			x							x													x						x
Ye and Griffin (2013)	1																							x						
Ye and Griffin (2016)	1																							x						
Yildiz and Güçlü (2013)	1																													x
Yildiz et al. (2015)	1																													x
Total	1	1	1	7	4	6	4	1	4	6	6	1	1	2	1	3	6	1	1	1	1	3	1	17	1	1	2	1	2	16

^1^Frequency varied between 1 and 1000 Hz

**Table 9:** Frequencies (> 250 Hz) at which VPT (or similar) were assessed

Reference	Total	Frequency (Hz)													
		261.6	300	320	392	400	500	523.3	600	700	784	800	900	1000	1046.5
Clemm et al. (2019)	1						x								
Dahlin et al. (2015)	1						x								
Ekman et al. (2019)	1						x								
Ekman et al. (2021)	1						x								
Flondell et al. (2017)	1						x								
Hatzfeld et al. (2016)	4			x			x							x	
Held et al. (2021)	2						x								
Hopkins et al. (2016) — Experiment I (fingertip)	5	x			x			x			x				x
Lundström et al. (2018)	1						x								
Prsa et al. (2021) — Detection Task in Humans	8		x			x	x		x	x		x	x	x	
Witte et al. (2022)	2						x								
Total		1	1	1	1	1	10	1	1	1	1	1	1	2	1

**Fig. 4:**
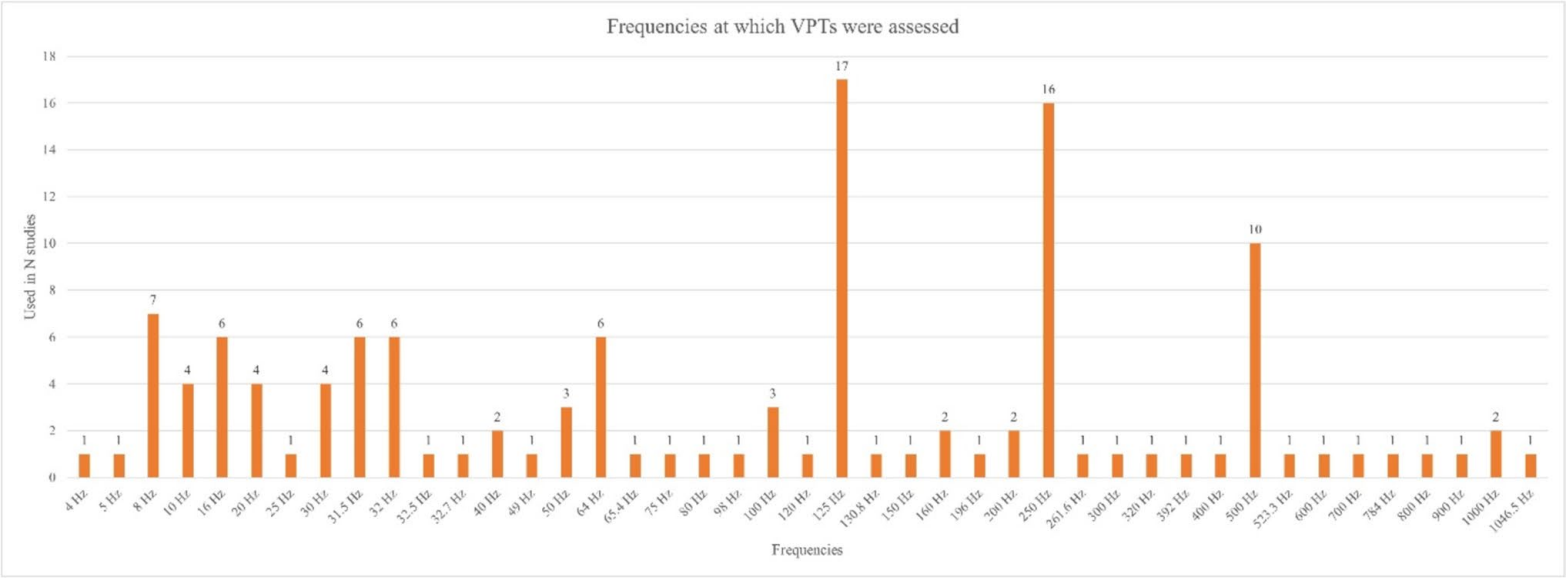
Frequencies at which VPTs were assessed across 38 studies

Across the 38 studies, VPTs were assessed for a total of 43 different frequencies. Most reviewed studies assessed VPT data only for one (15 studies) or two frequencies (11 studies). Six studies assessed VPTs for seven frequencies, and two studies for five frequencies. Lastly, solely one study assessed data for 3, 9, 11, and 14 different frequencies. Figure 4 presents all the different frequencies that were studied across the 38 studies, indicating in how many studies each of those frequencies were used to assess VPTs.

### Number of frequencies studied per hand location

**Fig. 5:**
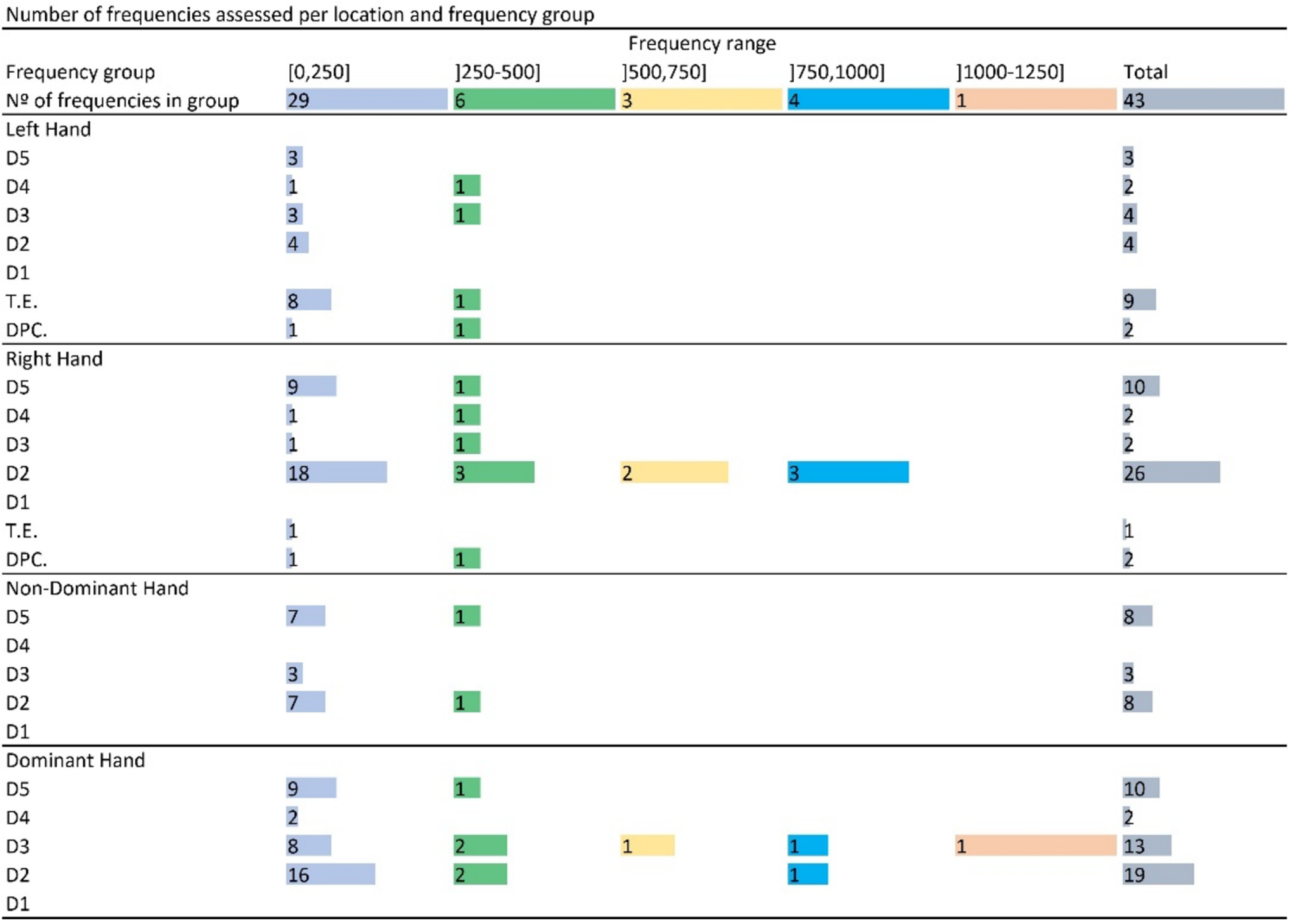
Number of frequencies at which VPTs were assessed per frequency range and hand location

Figure 5 presents an overview of how many different frequencies have been assessed per each hand location and frequency range (each range covering a span of 250 Hz, including). The Right Hand’s Index Finger (i.e., D2) is the area of the hand at which VPTs for the highest number of frequencies have been assessed (26 out of 43 frequencies). This location is followed by the Dominant Hand’s D2 (19 out of 43 frequencies) and Middle Finger (D3, 13 out of 43 frequencies). Conversely, the locations at which VPTs have been assessed for the lowest number of frequencies are the Right Hand’s Thenar Eminence (T.E., one frequency), Distal Palmar Flexion Crease (DPFC.), Ring Finger (D4), and D3 (two frequencies each), the Left Hand’s D4, DPFC, (two frequencies each) and Little Finger (D5, three frequencies), the Non-Dominant Hand’s D3 (three frequencies), and the Dominant Hand’s D4 (two frequencies).

**Fig. 6:**
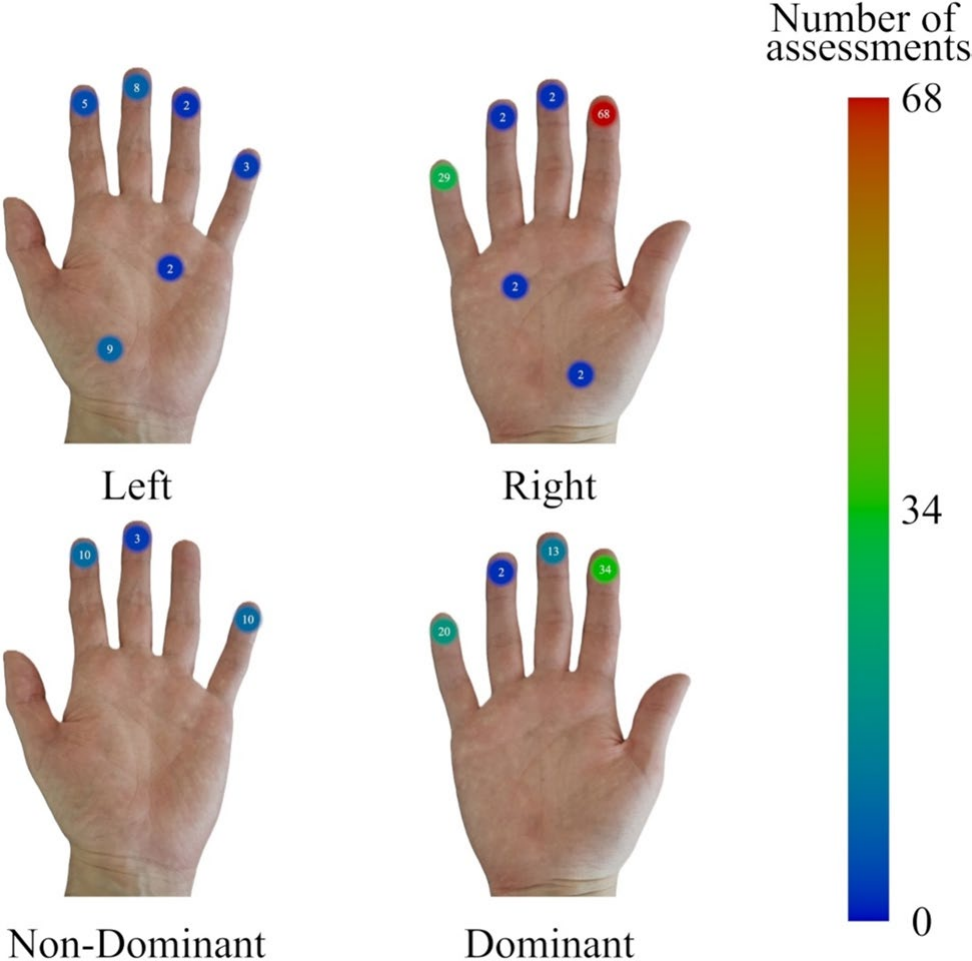
Heat map distribution of the number of VPT assessments per hand location

Figure 6, in turn, presents a heat map distribution of how many total assessments have been conducted at each hand location. To clarify, the number of participants with whom assessments were conducted in each paper was not considered for this figure. Instead, only the number of times that each combination of frequency and hand location was found across all reviewed papers, excluding Chauvelin et al. (2014), was tallied. During this process, in the case of studies that assessed VPTs at more than one point of a single area of the hand, each of those assessments was tallied once. For example, Yildiz and Güçlü (2013) assessed VPTs for a frequency of 250 Hz at three points of the Left Hand’s D3. These were tallied as three assessments for this combination of hand location (D3) and frequency (250 Hz). The information presented in Figs. 5 and 6 is given in more detail, according to each reviewed study, in Tables 2S and 3S, available as supplementary material.

## Discussion

This paper presented a systematic review, following the PRISMA 2020 methodology (Page et al., 2021), to answer the following question: "How are vibration perception thresholds assessed on the glabrous skin of the hands and fingers of healthy humans?". We gathered 3396 records from five of the main electronic databases in three different searches at three different dates, and the content of 39 papers was meticulously organized and analyzed to characterize the landscape of experimental practices used to assess vibration perception thresholds (VPTs) at the glabrous skin of the human hands and fingers.

### Study goals

From analyzing the goals of each of the 39 papers, and, more specifically, the goals pertaining to the studies that specifically assessed VPTs, it is evident that these assessments were utilized for various purposes. For example, some studies conducted VPT assessments to examine how aspects of the human body, like the presence of different pathologies, physical responses to different stimuli, or different physical characteristics, or aspects related to experimental protocols themselves, such as the presence of other stimuli, or different procedural methods, affect VPTs. Case in point, Oh and Choi (2019) studied the potential effect of the pressing force exerted between the skin location and the measurement instrument on the obtained VPT results. The contributions made by Oh and Choi (2019), among other studies, as shown in Table 3, are relevant to the field of study of human touch perception, as they report on the potential effects that different aspects of experimental protocols or different physical responses to external stimulation, for example, may have on VPT assessment results. Besides contributing to our knowledge regarding the internal mechanisms of human perception of vibration, results from these studies can help researchers improve upon the assessment procedures that are already in use by helping them be aware of the potential effect that different variables—such as vibration direction, or the amount of pressure between the probe and the skin (Pra et al., 2023), or the size of the probe itself (Gu & Griffin, 2013), or how wrinkly the skin at the assessment location is (Haseleu et al., 2014)—may have on VPTs assessed at the glabrous skin of the hand. By being aware of these variables, researchers can more easily identify which of them they might have to consider during their research, both during the planning stages (e.g., which variables to control for or monitor) and when reporting their methods and results.

Besides factors revolving around experimental procedures, other studies investigated the potential effect on VPTs of variables one might deal with throughout the day, such as physical responses of the body to outside stimuli, or the effects on the body of pathologies. For example, Tamrin et al. (2016) assessed the potential effects of chemical exposure on VPTs, while Held et al. (2021) compared the VPT results obtained from subjects with Dupuytren disease—a pathology that results in one or more fingers bending towards the palm—to those obtained from healthy comparison subjects. Results of studies conducted with clinical populations can be used to help medical professionals diagnose these pathologies better and earlier, promoting a more expedient treatment. VPT assessments can aid in the detection of the onset of various pathologies (Szabo et al., 1984; Thomsen et al., 2011). Additionally, information regarding VPTs and the factors that affect them can also be used by developers of human–machine interfaces (HMIs). For example, this information can help develop better prediction models of how vibrations might be perceived by users, based on the type of physical interactions that are intended to occur, how those vibrations will be generated, and the characteristics surrounding the entire interaction, from user to device components to environment.

### Participant population data

Regarding participant population data, we extracted the following information from the reviewed papers: country where the study occurred or participant’s reported nationality, sample size, gender distribution, overall age distribution, and distribution according to gender.

A wide variety of participant sample sizes, as well as a variety of participant characteristics, could be noted across the reviewed studies, as evident in Table 1S and mentioned in the “Participant population data” chapter of the “Methods” Section. This variety is potentially due to the different goals each study had. While the unbalanced gender distribution of some studies might be caused by, for example, a lack of female participants on the workforce that was the target of study (as might have occurred in Gerhardsson et al., 2013; and Lundström et al., 2018, for example), nonetheless, when looking at this data in a global manner, data from both male and female participants was gathered with an almost equal distribution (50.33% and 47.67%, respectively).

From the list of countries included in Table 1S and mentioned in the “Participant population data” chapter of the “Methods” Section, it is noticeable that the study of VPTs is carried out at various locations around the world. It is also noticeable that the populations of some countries (e.g., six studies each were conducted in Sweden and Japan) have been included in studies more often than populations from other countries (e.g., one study each was conducted in Malaysia, France, and Chile, among other countries).

According to Ahn et al. (2013), one of the limitations of the objective tests used to evaluate the presence of hand–arm vibration syndrome (HAVS), amongst which one can find the assessment of VPTs, is that their standards of diagnosis were created from Western populations' data. While it is not expected that a person’s nationality will have a direct influence on their VPTs, other factors related to one’s nationality or associated with the country in which a study is carried out—such as one’s race, or the region of the world in which they are found (Ahn et al., 2013)—may have some influence.

While gathering data from participants of other countries would be valuable, for example, to help solidify the currently existing normative data regarding VPTs for various frequencies and assess if the currently available normative data holds true for the population of these countries, this is, realistically speaking, largely dependent on various factors. One such factor is the presence of researchers interested in and willing to carry out these studies in these countries, as well as the existence of funding to support these efforts.

### Assessment instruments

It is interesting to note that about half of the studies that were reviewed (20 of 39) made use of custom-made apparatuses to assess VPTs, while a similar number of studies made use of commercially available devices (18 of 39). Commercial devices can be useful for various reasons in VPT studies. For one, data acquired from different studies that used the same instrument and model are theoretically easier to compare since different devices of the same model and version are expected to have the same specifications—such as probe size, frequencies employed, and available psychophysical algorithms. Thus, if other aspects of the experimental protocols, such as room temperature, are consistent across studies, their data can be more accurately compared, be it with other studies, or with available normative data collected from instruments with the same or similar characteristics.

However, these same manufacturer specifications may be hindrances to researchers who intend to conduct studies with variables that fall outside their specifications. One example of this potential limitation is the fact that, of the 18 studies that made use of commercially available instruments, 14 gathered VPT data using the von Békésy algorithm, while only one study did so with a custom-made apparatus. Throughout these 14 studies, the two most often used instruments were HVLab instruments, i.e., the HVLab Tactile Vibrometer and the HVLab Vibrotactile Perception Meter, used in five studies, and VibroSense Meter ® instruments, i.e., VibroSense Meter ® Version I and Version II, used in six studies. Analyzing the documentation that could be found for three of these four instruments (University of Southampton, n.d.-a, n.d.-b; VibroSense Dynamics AB, n.d.), excluding the VibroSense Meter ® Version I, it can be noted that all three only allow for data to be (natively) collected through the von Békésy algorithm.

When using custom instruments, in turn, preferences instead mainly fell on the use of the staircase method, used in seven of the 20 studies that employed a custom-made apparatus, with the two-answer or alternative forced-choice (2AFC) and the two-interval forced-choice (2IFC) task methods being the second and third most preferred, used in four and three studies, respectively (the latter tied with the use of the Method of Limits throughout these 20 studies). The Staircase, 2AFC, and 2IFC task methods, as described in each paper that employed them, made use of intermittent stimulation, which, according to ISO 13091–1 (International Organization for Standardization, 2001), is preferable to conduct VPT assessments over methods that use constant stimuli, such as the von Békésy algorithm. This preference is due to intermittent stimulation allowing subjects to not only contrast the applied vibrations against those naturally present in the background but also to limit the occurrence of temporary threshold shifts caused by subjects being exposed to stimuli higher than their thresholds, leading to more accurate threshold results. Nonetheless, one of the major limitations of the Staircase, 2AFC task, and 2IFC task methods is the substantial time needed for subjects to complete one set of assessments, especially when compared with the von Békésy method. In the von Békésy method, stimuli presentation is constant and continuous, meaning there are no time intervals between the actuation of different levels of stimuli; the measurements are also considerably faster. The time efficiency of assessing VPTs through the von Békésy method may be one of the motivators behind its prevalent use in commercial devices. As these are mostly intended for use by medical professionals to assess the presence or progress of various pathologies, professionals whose time during consultations is quite often limited, this aspect may have some effect during the development of commercial VPT assessment instruments, with developers preferring to implement a methodology that is both recommended under international standards, such as ISO 13091–1 (International Organization for Standardization, 2001), but also not very time-consuming.

Among the seven studies that employed the Staircase method and used a custom-made apparatus is the work of Chauvelin et al. (2014). While the usual approach to studying VPT is to manipulate stimuli amplitude to access the threshold level for a given frequency (as was done in the other 38 studies), these authors, instead, chose to manipulate stimuli frequency to access the threshold level for a given amplitude. One of the difficulties of this approach is that it requires a measuring instrument capable of maintaining similar vibration amplitude levels while frequencies change. Such a requirement might not be easily achieved with a commercial device and might be what led the authors to create their own apparatus. Thus, one can hypothesize that limitations such as these, brought about by researchers wanting to conduct studies following experimental designs that are less typical, may be the reason why so many authors choose to carry out VPTs studies using their own developed instruments.

As mentioned previously, another potential reason might be that researchers want to assess VPTs for variables outside commercial devices' specifications, such as frequencies other than those included in the commercial device’ settings. For example, the highest frequency from which VPTs were assessed using a custom-made apparatus was 1046.5 Hz, in Hopkins et al. (2016), while the highest frequency studied with a commercial device was 500 Hz (e.g., Dahlin et al., 2015; Ekman et al., 2019; Flondell et al., 2017), which is a huge difference. Thus, authors who value more flexibility in their experimental protocols, at least when it comes to the choice of which frequencies to assess VPTs from and which methodology to use for said assessments, will potentially have to either develop their own devices or adapt some existing ones.

Similarly, authors who wish to employ experimental conditions involving more than one active vibration stimulus may also be more inclined to develop their own instruments. According to the descriptions provided for all the commercial devices (devices identified in Table 4), none of these instruments can deliver vibration stimuli to multiple locations simultaneously. Thus, in studies where VPTs were assessed at different hand locations with these instruments, participants either had to move their hands over it or the instrument itself had to be moved so that the vibrating probe and the next skin location would meet. This, of course, results in participants expecting a vibration to be felt at said location during the next experimental conditions, and, if said conditions start by providing stimuli that is below expected threshold levels, it may lead to thresholds being lower than they would if no such expectations were held.

### Units of report

The creation of custom-made apparatuses, however, should not be taken lightly. When making such instruments, creators must ensure that they can not only collect VPT data accurately but also allow authors to report the acquired data using easily comparable and understandable metrics. Across the 39 studies, the most common metric to report VPTs were to: (1) either provide information regarding amplitude displacement in micrometers (µm), in 15 studies or (2) provide information regarding amplitude acceleration, reported in either decibels (dB) relative to 10^–6^ m/s^2^ (or dB re. 10^–6^ m/s^2^), as seen in 14 studies, or (3) as meters/second^2^ (m/s^2^; ms^–2^), as employed in five studies. Thus, authors would be advised to report their VPT results using at least one of these units of measurement. dB re. 10^–6^ m/s^2^ and m/s^2^, in particular, can be found in the ISO documents relevant to VPT assessments, namely ISO 13091–1 (International Organization for Standardization, 2001) and ISO 13091–2 (International Organization for Standardization, 2003). An added advantage of these three units of measurement (µm, dB re. 10^–6^ m/s^2^, and m/s^2^) is that, when provided alongside the necessary information—such as the reference value used to calculate dB, or the frequency of the vibration at which µm was acquired, and if they refer to either peak, peak-to-peak, or root-mean-square values—results expressed in said units can be converted to one another, making it easier to compare the results of multiple studies.

### Hand locations

Regarding the hand locations where assessments were conducted, it is noticeable that most studies (34 studies in total, or 87.18%) made assessments on the index finger (D2), irrespective of whether the assessments were conducted on the left, right, non-dominant, or dominant hands. The second most-assessed location, irrespective of hand, was the little finger (D5), in 38.46% (i.e., 15 studies), about half as many as the D2 (index). Conversely, the thumbs (D1) of the left, right, non-dominant, or dominant hands, were never the target of any assessments, while the distal palmar flexion crease (DPFC.) was only used for assessments in Held et al. (2021). This was also reflected in the total number of frequencies for which VPTs were assessed at these locations, meaning that substantially more information exists regarding VPTs for various frequencies at the D2, in contrast to all other locations of the hand.

This preference for conducting assessments at the D2 might reflect the fact that the index finger is the one most used for various activities, such as pointing or touching interactive displays (e.g., smartphones). This prominence throughout one’s day-to-day may influence researchers when they select at which hand locations to conduct assessments. However, it is important to keep in mind that, while some studies point to VPT results not being significantly different between different hand locations, or even between hands (e.g., Ahn et al., 2013; Dahlin et al., 2015; Koszewicz et al., 2021), others have found significant differences between VPTs at different hand locations, even if only at some frequencies (e.g., Ekman et al., 2021; Ye & Griffin, 2016), and potentially even at different points of the same hand location, such as the distal phalanx of a finger (Yildiz & Güçlü, 2013). This divergence in findings underscores the need for a more comprehensive approach to VPT assessments across separate locations. To this end, a suggestion would be for authors who are planning to conduct VPT assessments with less time-consuming methodologies, such as the von Békésy method or the method of limits, to collect data from more than one location of the same hand, accompanied whenever reasonable by the results of statistical tests examining if significant differences in VPT results could be found between such locations at whichever frequencies were tested. To facilitate comparisons with other studies, we would recommend that one of these locations be the index finger. Following this, whenever significant differences are found between the VPT results of different locations at certain frequencies, subsequent studies could then focus on collecting data from those locations and frequencies using more refined but time-consuming methodologies, such as the Staircase method or the 2AFC task, to further assess and potentially quantify differences found.

### Assessed frequencies

As for frequency, overall, it is particularly noticeable that all studies (38 studies, not including Chauvelin et al.,’s 2014 study, due to the referred divergent approach) have assessed VPTs for frequencies in the 0–250-Hz range, resulting in data being gathered for 29 different frequencies in this range. Comparatively, a much smaller number of studies (11 studies, 28.21%) have done so for frequencies in the 250–500-Hz range, with only six different frequencies being studied in this range, while even fewer (three studies, 7.69%) assessing VPTs for frequencies in the 500 Hz to > 1000 Hz, with VPT data being available for eight frequencies in this range.

While this difference between the amount of available data regarding VPTs for frequencies below and above 250 Hz might be due to valid reasons, such as a lack of access to tools that can accurately conduct assessments at frequencies higher than 250 Hz, or lack of motivation to carry out such assessments, it nonetheless results in gaps in our knowledge regarding how our brain perceives high-frequency vibrations. While it is not expected that the thresholds for all potential frequencies across all potential combinations of factors—such as subject age, skin temperature, and pressure between probe and skin, among many other variables—can one day be assessed, collecting data for more and more of these combinations can allow researchers to create more reliable and robust models to predict the VPTs for those combinations for which data are currently missing, so that said the information could be put to further use, be it, for example, in assessment studies to analyze if those predictions are indeed accurate, or to provide data to algorithms that control the vibrotactile feedback provided by various HMI devices.

### Review limitations and suggestions for future reviews

This review has also some limitations that should be considered. First, we only searched for and collected works published in the English language. However, research on VPTs has also been published in other languages, such as Polish (Harazin et al., 2004; Zamysłowska-Szmytke et al., 2002). Secondly, while our search terms of “vibration”, “perception”, and “threshold” were based on the well-established and widely used term “vibration perception threshold”, other terms, such as “vibration detection threshold”, “vibrotactile perception threshold”, and “vibrational perception threshold,” were also encountered on some papers on this topic during our analysis. While these papers were given the same attention as all others on this topic during our analysis, future authors could opt to employ these search terms and/or translations of these terms into other languages as well, and/or opt for reviewing papers published over a wider period, to further broaden the scope of their reviews. Furthermore, said future authors may also opt to include records obtained from electronic databases other than the ones through which we conducted searches for this review, such as the Online Knowledge Library (b-on), to broaden the scope of their reviews further.

Another limitation still is in regards to the *C.Metrics* criterion. While the results from each of the collected papers were not focused on for this review’s analysis, we nevertheless made efforts to only include papers that presented concrete numerical quantitative results from their assessments, accompanied by detailed information regarding the units of measurement used to report those same results. Thus, papers that presented their results in relative units but without a clear disclosure of which values were relative to, were considered to not meet this criterion. This approach was taken to only provide readers with papers whose results could be collected, analyzed, and compared with results from other studies. This practice not only hampers the advancement of the field by making cross-study comparisons difficult or impossible, but it also particularly affects readers who are less familiar with the field. These readers may struggle to interpret the various measurement units used in VPTs assessments over time, potentially leading to misinterpretations of the results. In a similar vein, papers that only reported their VPT results in terms of how much they deviated from other data (e.g., deviations from normative data) were also not considered to meet the *C.Metrics* criterion, as not all readers can be expected to have access to all available normative data, thus complicating the matter of analyzing results. Although considering these aspects limited the number of papers included in this review, our aim was also that this review could serve as a jumping point for readers wishing to dive further into their own analysis of the VPT results of the papers that caught their interest.

### Final remarks

One of the most important findings of our review is that, when paired up, the difference between the number of frequencies studied below 250 Hz in contrast to those above 250 Hz, and the lack of VPT data for certain hand locations, further increases the gaps in our knowledge, more so as there is still a debate regarding if different hand locations share some of the same thresholds or not. Thus, more research would be welcomed to address these gaps, including studies involving VPT assessments for other frequencies that have been less (or never) studied before in the published literature. VPT assessments for different frequencies in less studied hand locations, as well as VPT studies involving populations that have less often been the subject of study (e.g., children or senior adults), would help solidify and improve the normative data that is currently available for various clinical diagnostical purposes.

This information could also prove beneficial in developing human–machine interfaces that better use vibrotactile feedback. By acquiring more information, developers' confidence in their designs may be improved, as they will be better equipped to base their vibrotactile interactions on concrete scientific data. If said interactions are likely or intended to occur in environments in which users are subjected to background vibrations, which may mask the intentional vibrotactile warnings and signals—e.g., when riding a motorcycle vehicle (Lisboa et al., 2024)—the use of vibrations of higher frequencies might be a worthwhile alternative, as such frequencies may be noticeable in the noisy background. Such an alternative would nonetheless require VPT data for said higher frequency vibrations to be studied, to check not only the viability of its implementation but also how different higher frequencies could potentially be used to improve the expressiveness of vibrotactile feedback during different interaction events.

While this paper focused on acquiring the experimental practices employed to assess VPTs at the human hands and fingers in the literature, VPT assessments have also been conducted in other areas of the body, such as the non-glabrous (hairy) skin of the hand, the arms or back. Further systematic reviews poring over this information may also prove useful for both researchers and designers, as such information can help shape future advancements in both research and technology. Another potential subject for a systematic review would be to, contrary to what was done in this paper, focus on VPT information acquired for non-healthy subjects, as such data may also prove useful for medical endeavors. Furthermore, future works such as these could also pour over studies published in languages other than English, as the studies included in this review were included based on this language restriction.

## Conclusion

The scientific community is investigating factors that influence our perception of vibration in our hands. This includes determining the minimum amount of vibration amplitude required for conscious perception. However, due to the large body of work that has come beforehand, it may be difficult for researchers to be aware of what has and has not been studied before. This review intended to mitigate this by shedding light on some aspects regarding the experimental practices of said works, such as which hand locations, frequencies, experimental methodologies, and instruments, have been used throughout the literature. We greatly contributed to increasing the knowledge of how humans perceive vibrations by identifying gaps and proposing future directions for a more comprehensive understanding of human haptic perception.

## Supplementary Information

Below is the link to the electronic supplementary material.

Supplementary file1 (DOCX 96 KB)

## Data Availability

All data related to this systematic review is openly available at the Open Science Framework (OSF) at the following URL: https://osf.io/zupxa/?view_only=b883bc72e8d74553876e786d339d3319.

## Declarations

### Open practices statement

Data and materials for this systematic review are openly available at https://osf.io/zupxa/?view_only=b883bc72e8d74553876e786d339d3319. This review was not preregistered.
