# Supplementary material for: How to determine hands’ vibration perception thresholds – a systematic review
Source: Behav Res Methods. 2024 Dec 28;57(1):27. doi: 10.3758/s13428-024-02534-w (PMC11682013; doi:10.3758/s13428-024-02534-w)
Supplement: Supplementary file 1 — Supplementary file1 (DOCX 96 KB) [file 13428_2024_2534_MOESM1_ESM.docx]

**Search strings used in each database, and number of entries returned in each**

**2012-2022 search**

- Scopus
  - “[TITLE-ABS-KEY ( ( vibration AND perception AND threshold ) AND ( hand OR finger ) ) AND PUBYEAR > 2011 AND PUBYEAR < 2023 AND ( LIMIT-TO ( LANGUAGE , "English" ) )](https://www.scopus.com/results/results.uri?sort=plf-f&src=s&sid=1b5d3d798e11708723a5da26ede9f656&sot=a&sdt=a&cluster=scolang%2C%22English%22%2Ct&sl=127&s=TITLE-ABS-KEY+%28+%28+vibration+AND+perception+AND+threshold+%29+AND+%28+hand+OR+finger+%29+%29+AND+PUBYEAR+%26gt%3B+2011+AND+PUBYEAR+%26lt%3B+2023&origin=searchadvanced&editSaveSearch=&txGid=aef6d5add1dc8d04ed78acbe9749f7da&sessionSearchId=1b5d3d798e11708723a5da26ede9f656&limit=200)”, with 185 results.
  - “[TITLE-ABS-KEY ( "Vibration perception thresholds" AND finger ) AND PUBYEAR > 2011 AND PUBYEAR < 2023 AND ( LIMIT-TO ( LANGUAGE , "English" ) )](https://www.scopus.com/results/results.uri?sort=plf-f&src=s&sid=e16b0c5b7e07786777ffcecd5d1f4cf4&sot=a&sdt=a&cluster=scolang%2C%22English%22%2Ct&sl=103&s=TITLE-ABS-KEY%28%22Vibration+perception+thresholds%22+AND+finger%29+AND+PUBYEAR+%26gt%3B+2011+AND+PUBYEAR+%26lt%3B+2023&origin=searchadvanced&editSaveSearch=&txGid=857239e96fcbaa7e7f385b83a763fa8a&sessionSearchId=e16b0c5b7e07786777ffcecd5d1f4cf4&limit=10)”, with 20 results.
  - “[TITLE-ABS-KEY ( "Vibration perception thresholds" AND hand ) AND PUBYEAR > 2011 AND PUBYEAR < 2023 AND ( LIMIT-TO ( LANGUAGE , "English" ) )](https://www.scopus.com/results/results.uri?sort=plf-f&src=s&sid=1fd5695ab9d7ca0863bd527591c45053&sot=a&sdt=a&cluster=scolang%2C%22English%22%2Ct&sl=101&s=TITLE-ABS-KEY%28%22Vibration+perception+thresholds%22+AND+hand%29+AND+PUBYEAR+%26gt%3B+2011+AND+PUBYEAR+%26lt%3B+2023&origin=searchadvanced&editSaveSearch=&txGid=e609c46223f685932e50050db9b9975f&sessionSearchId=1fd5695ab9d7ca0863bd527591c45053&limit=100)”, with 35 results.
- Web of Science (WoS)
  - “[(ALL=((vibration AND perception AND threshold))) AND ALL=((Hand OR Finger)) and English (Languages)](https://www.webofscience.com/wos/woscc/summary/bb65581f-df0f-4f43-becd-cb6c69a6a627-67f04447/relevance/1)”, with 165 results.
  - “[(ALL=(("Vibration perception thresholds"))) AND ALL=((Hand)) and English (Languages)](https://www.webofscience.com/wos/woscc/summary/5ba92cc4-cc7d-45c4-9740-be734356520c-6a6cf338/relevance/1)”, with 19 results.
  - “[(ALL=(("Vibration perception thresholds"))) AND ALL=((Finger)) and English (Languages)](https://www.webofscience.com/wos/woscc/summary/decc5b6c-0bd2-45aa-804b-9d10604610a1-6a6d6cf7/relevance/1)”, with 13 results.
- PubMed
  - “[Search: (( vibration AND perception AND threshold )) AND (( hand OR finger )) Filters: English, from 2012/1/1 - 2022/12/31](https://pubmed.ncbi.nlm.nih.gov/?term=%28%28+vibration+AND+perception+AND+threshold+%29%29+AND+%28%28+hand+OR+finger+%29%29&filter=dates.2012%2F1%2F1-2022%2F12%2F31&filter=lang.english)”, with 181 results.
  - “[Search: (( vibration AND perception AND threshold )) AND (( finger )) Filters: English, from 2012/1/1 - 2022/12/31](https://pubmed.ncbi.nlm.nih.gov/?term=%28%28+vibration+AND+perception+AND+threshold+%29%29+AND+%28%28+finger+%29%29&filter=dates.2012%2F1%2F1-2022%2F12%2F31&filter=lang.english&sort=relevance)”, with 99 results.
  - “[Search: (( vibration AND perception AND threshold )) AND (( hand )) Filters: English, from 2012/1/1 - 2022/12/31](https://pubmed.ncbi.nlm.nih.gov/?term=%28%28+vibration+AND+perception+AND+threshold+%29%29+AND+%28%28hand%29%29&filter=dates.2012%2F1%2F1-2022%2F12%2F31&filter=lang.english)”, with 168 results.

**2023 search**

- Scopus
  - “[TITLE-ABS-KEY ( ( vibration AND perception AND threshold ) AND ( hand OR finger ) ) AND PUBYEAR > 2022 AND PUBYEAR < 2024 AND ( LIMIT-TO ( LANGUAGE , "English" ) )](https://www.scopus.com/results/results.uri?sort=plf-f&src=s&sid=663cb1c0582cdcf9d11c302b2b8cbfef&sot=a&sdt=a&cluster=scolang%2C%22English%22%2Ct&sl=127&s=TITLE-ABS-KEY+%28+%28+vibration+AND+perception+AND+threshold+%29+AND+%28+hand+OR+finger+%29+%29+AND+PUBYEAR+%26gt%3B+2022+AND+PUBYEAR+%26lt%3B+2024&origin=searchadvanced&editSaveSearch=&txGid=2ca7dffd1fdd2ce552cae08b9792fb46&sessionSearchId=663cb1c0582cdcf9d11c302b2b8cbfef&limit=10)”, with 14 results.
  - “[TITLE-ABS-KEY ( "Vibration perception thresholds" AND finger ) AND PUBYEAR > 2022 AND PUBYEAR < 2024 AND ( LIMIT-TO ( LANGUAGE , "English" ) )](https://www.scopus.com/results/results.uri?sort=plf-f&src=s&sid=d6d4fff45821634a19c11688e218aa8b&sot=a&sdt=a&cluster=scolang%2C%22English%22%2Ct&sl=103&s=TITLE-ABS-KEY%28%22Vibration+perception+thresholds%22+AND+finger%29+AND+PUBYEAR+%26gt%3B+2022+AND+PUBYEAR+%26lt%3B+2024&origin=searchadvanced&editSaveSearch=&txGid=3a0ff1c2c4a7dfbc6ab2c9348a8aaa95&sessionSearchId=d6d4fff45821634a19c11688e218aa8b&limit=10)”, with 5 results.
  - “[TITLE-ABS-KEY ( "Vibration perception thresholds" AND hand ) AND PUBYEAR > 2022 AND PUBYEAR < 2024 AND ( LIMIT-TO ( LANGUAGE , "English" ) )](https://www.scopus.com/results/results.uri?sort=plf-f&src=s&sid=163e7306673c44b4eb85744d1ab00e7f&sot=a&sdt=a&cluster=scolang%2C%22English%22%2Ct&sl=101&s=TITLE-ABS-KEY%28%22Vibration+perception+thresholds%22+AND+hand%29+AND+PUBYEAR+%26gt%3B+2022+AND+PUBYEAR+%26lt%3B+2024&origin=searchadvanced&editSaveSearch=&txGid=33ece304d968b2efea4af94466b9e6fa&sessionSearchId=163e7306673c44b4eb85744d1ab00e7f&limit=10)”, with 5 results.
- Web of Science
  - “[(ALL=((vibration AND perception AND threshold))) AND ALL=((Hand OR Finger)) and English (Languages)](https://www.webofscience.com/wos/woscc/summary/a51b507c-7af3-49a6-b05b-e0f31a4d1d9e-b7734afe/relevance/1)”, with 5 results.
  - “[(ALL=(("Vibration perception thresholds"))) AND ALL=((Hand)) and English (Languages)](https://www.webofscience.com/wos/woscc/summary/7607053f-5fa7-44fe-93cb-940cc0d9d172-b77384a5/relevance/1)”, with 1 results.
  - “[(ALL=(("Vibration perception thresholds"))) AND ALL=((Finger)) and English (Languages)](https://www.webofscience.com/wos/woscc/summary/ef47437c-f03e-4fa1-baa1-bfe9a80e93ce-b773ace1/relevance/1)”, with 0 results.
- PubMed
  - “[Search: (( vibration AND perception AND threshold )) AND (( hand OR finger )) Filters: English, from 2023/1/1 - 2023/12/31](https://pubmed.ncbi.nlm.nih.gov/?term=((%20vibration%20AND%20perception%20AND%20threshold%20))%20AND%20((%20hand%20OR%20finger%20))&filter=dates.2023%2F1%2F1-2023%2F12%2F31&filter=lang.english)”, with 9 results.
  - “[Search: (( vibration AND perception AND threshold )) AND (( finger )) Filters: English, from 2023/1/1 - 2023/12/31](https://pubmed.ncbi.nlm.nih.gov/?term=%28%28+vibration+AND+perception+AND+threshold+%29%29+AND+%28%28+finger+%29%29&filter=dates.2023%2F1%2F1-2023%2F12%2F31&filter=lang.english)”, with 5 results.
  - “[Search: (( vibration AND perception AND threshold )) AND (( hand )) Filters: English, from 2023/1/1 - 2023/12/31](https://pubmed.ncbi.nlm.nih.gov/?term=%28%28+vibration+AND+perception+AND+threshold+%29%29+AND+%28%28hand%29%29&filter=dates.2023%2F1%2F1-2023%2F12%2F31&filter=lang.english)”, with 7 results.

**2012-2023 search**

- IEEE Xplore
  - “[(( vibration AND perception AND threshold ) AND ( hand OR finger ))](https://ieeexplore.ieee.org/search/searchresult.jsp?action=search&matchBoolean=true&queryText=(((%20vibration%20AND%20perception%20AND%20threshold%20)%20AND%20(%20hand%20OR%20finger%20)))&highlight=true&returnType=SEARCH&matchPubs=true&ranges=2012_2023_Year&returnFacets=ALL)”, with 80 results.
  - “[(( "Vibration perception thresholds" ) AND ( finger))](https://ieeexplore.ieee.org/search/searchresult.jsp?action=search&matchBoolean=true&newsearch=true&queryText=(((%20%22Vibration%20perception%20thresholds%22%20)%20AND%20(%20hand%20))))”, with 3 results.
  - “[(( "Vibration perception thresholds" ) AND ( hand ))](https://ieeexplore.ieee.org/search/searchresult.jsp?action=search&matchBoolean=true&newsearch=true&queryText=(((%20%22Vibration%20perception%20thresholds%22%20)%20AND%20(%20hand%20))))”, with 3 results.
- ACM Digital Library
  - “[{ ( vibration AND perception AND threshold ) AND ( hand OR finger ) }](https://dl.acm.org/action/doSearch?fillQuickSearch=false&target=advanced&expand=dl&AfterMonth=1&AfterYear=2012&BeforeMonth=12&BeforeYear=2023&AllField=%28+vibration+AND+perception+AND+threshold+%29+AND+%28+hand+OR+finger+%29)”, with 2368 results.
  - “[( "vibration perception threshold" ) AND ( finger )](https://dl.acm.org/action/doSearch?fillQuickSearch=false&target=advanced&expand=dl&AfterMonth=1&AfterYear=2012&BeforeMonth=12&BeforeYear=2023&AllField=%28+%22vibration+perception+threshold%22+%29+AND+%28+finger+%29)”, with 4 results.
  - “[( "vibration perception threshold" ) AND ( hand )](https://dl.acm.org/action/doSearch?fillQuickSearch=false&target=advanced&expand=dl&AfterMonth=1&AfterYear=2012&BeforeMonth=12&BeforeYear=2023&AllField=%28+%22vibration+perception+threshold%22+%29+AND+%28+hand+%29)”, with 2 results.

*Table 1S — Characterization of each study's healthy/control participant population.*

| **Reference** | **Country** | **Nº** | **Nº Male** | **Nº Female** | **Age (overall), y/o** | | | | | **Age (male), y/o** | | | | | **Age (female), y/o** | | | | |
| --- | --- | --- | --- | --- | --- | --- | --- | --- | --- | --- | --- | --- | --- | --- | --- | --- | --- | --- | --- |
|  |  |  |  |  | **M** | **SD** | **IQR** | **Min.** | **Max.** | **M** | **SD** | **IQR** | **Min.** | **Max.** | **M** | **SD** | **IQR** | **Min.** | **Max.** |
| Ahn et al. (2013) | KR | 120 | 120 | 0 |  |  |  |  |  |  |  |  | 30 | 59 | N/A | N/A | N/A | N/A | N/A |
| Arredondo and Perez (2017) — Experiment 1 | Chile | 13 | 12 | 1 |  |  |  | 22 | 39 |  |  |  |  |  |  |  |  |  |  |
| Calder et al. (2012) | Canada | 9 | 0 | 9 |  |  |  |  |  | N/A | N/A | N/A | N/A | N/A | 56.67 | 5.5 |  |  |  |
| Chauvelin et al. (2014) | France | 46 | 23 | 23 | 39.6 | 10 |  | 21 | 60 |  |  |  |  |  |  |  |  |  |  |
| Clemm et al. (2019) | Norway | 19 |  |  | 33.7 | 11.1 |  |  |  |  |  |  |  |  |  |  |  |  |  |
| Dahlin et al. (2015) | Sweden | 269 | 130 | 139 |  |  |  | 8 | 20 |  |  |  |  |  |  |  |  |  |  |
| Ekman et al. (2019) | Sweden | 20 | 10 | 10 | 46 | 11.1 |  | 26 | 65 |  |  |  |  |  |  |  |  |  |  |
| Ekman et al. (2021) | Sweden | 913 | 293 | 620 | 46 |  |  | 18 | 90 |  |  |  |  |  |  |  |  |  |  |
| Flondell et al. (2017) | Sweden | 66 | 16 | 50 | 46.5 |  | 11 |  |  |  |  |  |  |  |  |  |  |  |  |
| Folmli et al. (2018) | Australia | 29 | 15 | 14 | 22.86 | 6.78 |  |  |  |  |  |  |  |  |  |  |  |  |  |
| Experimental group |  | 14 |  |  |  |  |  |  |  |  |  |  |  |  |  |  |  |  |  |
| Sham group |  | 15 |  |  |  |  |  |  |  |  |  |  |  |  |  |  |  |  |  |
| Gerhardsson et al. (2013) | Sweden | 41 | 41 | 0 |  |  |  |  |  | 20.7 | 0.9 |  |  |  | N/A | N/A | N/A | N/A | N/A |
| Gu and Griffin (2013) | U.K. | 12 | 12 | 0 |  |  |  |  |  | 27 |  |  |  |  | N/A | N/A | N/A | N/A | N/A |
| Güçlü and Dinçer (2013) | Turkey | 8 | 4 | 4 |  |  |  |  |  | 25.5 |  |  | 22 | 29 | 24.2 |  |  | 23 | 26 |
| Haseleu et al. (2014) | Germany | 20 | 8 | 12 | 27.9 | 2.3 |  | 20 | 30 |  |  |  |  |  |  |  |  |  |  |
| Hatzfeld et al. (2016) | Germany | 27 | 17 | 10 | 24 | 2 |  | 21 | 31 |  |  |  |  |  |  |  |  |  |  |
| Held et al. (2021) | U.S.A. | 74 | 28 | 46 | 62.07 | 11.41 |  | 18 | 76 | 62.61 |  |  |  |  | 61.74 |  |  |  |  |
| Hopkins et al. (2016) — Experiment I (fingertip) | U.K. | 32 | 19 | 13 | 30.6 | 9.2 |  | 18 | 65 |  |  |  |  |  |  |  |  |  |  |
| Koszewicz et al. (2021) | Poland | 31 | 14 | 17 | 44.9 | 15.5 |  |  |  | 42.8 |  |  |  |  | 46.7 |  |  |  |  |
| Kowalski and Zając (2012) | Poland | 10 | 10 | 0 |  |  |  |  |  | 31.2 | 9.1 |  | 24 | 52 | N/A | N/A | N/A | N/A | N/A |
| Labbé et al. (2016) — Vibrotactile Detection Task | Canada | 13 | 4 | 9 | 22 |  |  | 18 | 25 |  |  |  |  |  |  |  |  |  |  |
| Lundström et al. (2018) | Sweden | 33 | 33 | 0 |  |  |  |  |  | 44.4 | 11.7 |  | 23 | 64 | N/A | N/A | N/A | N/A | N/A |
| Marcuzzi et al. (2019) | Australia | 50 | 25 | 25 | 37 | 11.5 |  | 20 | 60 |  |  |  |  |  |  |  |  |  |  |
| Moshourab et al. (2017) | Germany | 111 | 53 | 58 | 16 |  |  | 14 | 19 |  |  |  |  |  |  |  |  |  |  |
| Niwa et al. (2021) — Experiment I | Japan | 8 | 7 | 1 |  |  |  | 22 | 23 |  |  |  | 22 | 23 |  |  |  | 22 | 23 |
| Oh and Choi (2019) | KR | 10 | 7 | 3 | 23.9 | 2.8 |  | 19 | 29 |  |  |  |  |  |  |  |  |  |  |
| Papetti et al. (2017) | — | 27 |  |  | 26 | 4.5 |  | 19 | 39 |  |  |  |  |  |  |  |  |  |  |
| Pra et al. (2022) | Italy | 22 | 15 | 7 | 31 | 7.8 |  |  |  |  |  |  |  |  |  |  |  |  |  |
| Prsa et al. (2021) — Detection Task in Humans | Switzerland | 19 | 10 | 9 | 30.21 | 8.38 |  |  |  |  |  |  |  |  |  |  |  |  |  |
| Sakurai and Shinoda (2014) — ES Device Evaluation Experiment | Japan | 7 | 7 | 0 |  |  |  |  |  |  |  |  | 21 | 32 | N/A | N/A | N/A | N/A | N/A |
| Shibata (2022) | Japan | 20 | 20 | 0 |  |  |  |  |  |  |  |  | 20 | 75 | N/A | N/A | N/A | N/A | N/A |
| Group 2 (G2) |  | 10 | 10 | 0 |  |  |  |  |  | 70.8 | 3.3 |  | 66 | 75 | N/A | N/A | N/A | N/A | N/A |
| Group 3 (G3) |  | 10 | 10 | 0 |  |  |  |  |  | 21.8 | 1.5 |  | 20 | 24 | N/A | N/A | N/A | N/A | N/A |
| Shibata (2023) | Japan | 20 | 20 | 0 |  |  |  | 20 | 75 |  |  |  | 20 | 75 |  |  |  |  |  |
| Group 2 (G2) |  |  | 10 |  |  |  |  |  |  | 70.8 | 3.3 |  | 66 | 75 |  |  |  |  |  |
| Group 3 (G3) |  |  | 10 |  |  |  |  |  |  | 21.8 | 1.5 |  | 20 | 24 |  |  |  |  |  |
| Tamrin et al. (2016) | Malaysia | 120 | 120 | 0 |  |  |  |  |  | 28.4 |  |  |  | N/A | N/A | N/A | N/A | N/A | N/A |
| Tanaka et al. (2015) - Detection Experiment | Japan | 14 | 9 | 5 | 20.9 |  |  | 19 | 24 |  |  |  |  |  |  |  |  |  |  |
| Tanaka et al. (2016) | Japan | 16 | 13 | 3 | 20.6 |  |  | 18 | 26 |  |  |  |  |  |  |  |  |  |  |
| Witte et al. (2022) | Germany | 8 | 8 | 0 |  |  |  |  |  | 27.3 | 4 |  | 23 | 34 | N/A | N/A | N/A | N/A | N/A |
| Ye and Griffin (2013) | U.K. | 15 | 15 | 0 |  |  |  |  |  | 24.5 |  |  |  |  | N/A | N/A | N/A | N/A | N/A |
| Ye and Griffin (2016) | U.K. | 12 | 12 | 0 |  |  |  |  |  | 25.6 |  |  |  |  | N/A | N/A | N/A | N/A | N/A |
| Yildiz and Güçlü (2013) | Turkey | 7 | 3 | 4 |  |  |  | 23 | 30 |  |  |  |  |  |  |  |  |  |  |
| Yildiz et al. (2015) | Turkey | 10 | 5 | 5 | 27.8 |  |  | 25 | 33 |  |  |  |  |  |  |  |  |  |  |
| M = Medium; SD = Standard Deviation; IQR = Interquartile range; Min. = Minimum; Max. = Maximum; — = Not mentioned or specified; N/A = Nonapplied; U.K. = United Kingdom; KR = South Korea. | | | | | | | | | | | | | | | | | | | |

*Table 2S — Number of VPT assessments conducted, per Hand Location and Frequency (≤ 250 Hz).*

| Reference | Frequency (Hz) | | | | | | | | | | | | | | | | | | | | | | | | | | | | |
| --- | --- | --- | --- | --- | --- | --- | --- | --- | --- | --- | --- | --- | --- | --- | --- | --- | --- | --- | --- | --- | --- | --- | --- | --- | --- | --- | --- | --- | --- |
|  | 4 | 5 | 8 | 10 | 16 | 20 | 25 | 30 | 31.5 | 32 | 32.5 | 32.7 | 40 | 49 | 50 | 64 | 65.4 | 75 | 80 | 98 | 100 | 120 | 125 | 130.8 | 150 | 160 | 196 | 200 | 250 |
| **Left Hand** |  |  |  |  |  |  |  |  |  |  |  |  |  |  |  |  |  |  |  |  |  |  |  |  |  |  |  |  |  |
| D5 |  |  |  |  |  |  |  |  | 1 |  |  |  |  |  |  |  |  |  |  |  | 1 |  | 1 |  |  |  |  |  |  |
| D4 |  |  |  |  |  |  |  |  |  |  |  |  |  |  |  |  |  |  |  |  |  |  |  |  |  |  |  |  | 1 |
| D3 |  |  |  |  |  | 1 |  |  |  |  |  |  | 1 |  |  |  |  |  |  |  |  |  |  |  |  |  |  |  | 5 |
| D2 |  |  |  |  |  |  |  |  | 1 |  |  |  |  |  |  |  |  |  |  |  | 1 | 2 | 1 |  |  |  |  |  |  |
| D1 |  |  |  |  |  |  |  |  |  |  |  |  |  |  |  |  |  |  |  |  |  |  |  |  |  |  |  |  |  |
| T.E. |  |  | 1 |  | 1 | 1 |  |  |  | 1 |  |  |  |  |  | 1 |  |  |  |  |  |  | 1 |  |  | 1 |  |  | 1 |
| DPC. |  |  |  |  |  |  |  |  |  |  |  |  |  |  |  |  |  |  |  |  |  |  |  |  |  |  |  |  | 1 |
| **Right Hand** |  |  |  |  |  |  |  |  |  |  |  |  |  |  |  |  |  |  |  |  |  |  |  |  |  |  |  |  |  |
| D5 |  |  | 4 |  | 3 |  |  |  | 1 | 3 | 1 |  |  |  |  | 3 |  |  |  |  | 1 |  | 5 |  |  |  |  |  | 4 |
| D4 |  |  |  |  |  |  |  |  |  |  |  |  |  |  |  |  |  |  |  |  |  |  |  |  |  |  |  |  | 1 |
| D3 |  |  |  |  |  |  |  |  |  |  |  |  |  |  |  |  |  |  |  |  |  |  |  |  |  |  |  |  | 1 |
| D2 | 1 |  | 5 | 2 | 4 | 1 | 1 | 2 | 4 | 4 | 1 |  |  |  | 1 | 4 |  | 1 |  |  | 3 | 2 | 11 |  |  |  |  | 1 | 6 |
| D1 |  |  |  |  |  |  |  |  |  |  |  |  |  |  |  |  |  |  |  |  |  |  |  |  |  |  |  |  |  |
| T.E. |  |  |  |  |  |  |  |  |  |  |  |  |  |  |  |  |  |  |  |  |  |  | 2 |  |  |  |  |  |  |
| DPC. |  |  |  |  |  |  |  |  |  |  |  |  |  |  |  |  |  |  |  |  |  |  |  |  |  |  |  |  | 1 |
| **Non-Dominant Hand** |  |  |  |  |  |  |  |  |  |  |  |  |  |  |  |  |  |  |  |  |  |  |  |  |  |  |  |  |  |
| D5 |  |  | 1 |  | 1 |  |  |  | 1 | 1 |  |  |  |  |  | 1 |  |  |  |  |  |  | 2 |  |  |  |  |  | 2 |
| D4 |  |  |  |  |  |  |  |  |  |  |  |  |  |  |  |  |  |  |  |  |  |  |  |  |  |  |  |  |  |
| D3 |  |  |  |  |  |  |  | 1 |  |  |  |  |  |  |  |  |  |  |  |  |  |  |  |  |  |  |  | 1 | 1 |
| D2 |  |  | 1 |  | 1 |  |  |  | 1 | 1 |  |  |  |  |  | 1 |  |  |  |  |  |  | 2 |  |  |  |  |  | 2 |
| **Dominant Hand** |  |  |  |  |  |  |  |  |  |  |  |  |  |  |  |  |  |  |  |  |  |  |  |  |  |  |  |  |  |
| D5 |  |  | 2 | 1 | 2 |  |  |  | 1 | 2 |  |  |  |  | 1 | 2 |  |  |  |  |  |  | 4 |  |  |  |  |  | 3 |
| D4 |  |  |  |  |  |  |  |  | 1 |  |  |  |  |  |  |  |  |  |  |  |  |  | 1 |  |  |  |  |  |  |
| D3 |  |  |  |  |  |  |  | 1 |  |  |  | 1 |  | 1 |  |  | 1 |  |  | 1 |  |  |  | 1 |  |  | 1 | 1 |  |
| D2 |  | 1 | 2 | 1 | 2 | 1 |  | 1 | 1 | 2 |  |  | 1 |  | 2 | 2 |  |  | 1 |  |  |  | 3 |  | 1 | 1 |  |  | 7 |
| **Total** | 1 | 1 | 16 | 4 | 14 | 4 | 1 | 5 | 12 | 14 | 2 | 1 | 2 | 1 | 4 | 14 | 1 | 1 | 1 | 1 | 6 | 4 | 33 | 1 | 1 | 2 | 1 | 3 | 36 |

*Table 3S — Number of VPT assessments conducted, per Hand Location and Frequency (> 250 Hz).*

| Reference | Frequency (Hz) | | | | | | | | | | | | | |
| --- | --- | --- | --- | --- | --- | --- | --- | --- | --- | --- | --- | --- | --- | --- |
|  | 261.6 | 300 | 320 | 392 | 400 | 500 | 523.3 | 600 | 700 | 784 | 800 | 900 | 1000 | 1046.5 |
| **Left Hand** |  |  |  |  |  |  |  |  |  |  |  |  |  |  |
| D4 |  |  |  |  |  | 1 |  |  |  |  |  |  |  |  |
| D3 |  |  |  |  |  | 1 |  |  |  |  |  |  |  |  |
| T.E. |  |  |  |  |  | 1 |  |  |  |  |  |  |  |  |
| DPC. |  |  |  |  |  | 1 |  |  |  |  |  |  |  |  |
| **Right Hand** |  |  |  |  |  |  |  |  |  |  |  |  |  |  |
| D5 |  |  |  |  |  | 4 |  |  |  |  |  |  |  |  |
| D4 |  |  |  |  |  | 1 |  |  |  |  |  |  |  |  |
| D3 |  |  |  |  |  | 1 |  |  |  |  |  |  |  |  |
| D2 |  | 1 |  |  | 1 | 7 |  | 1 | 1 |  | 1 | 1 | 1 |  |
| DPC. |  |  |  |  |  | 1 |  |  |  |  |  |  |  |  |
| **Non-Dominant Hand** |  |  |  |  |  |  |  |  |  |  |  |  |  |  |
| D5 |  |  |  |  |  | 1 |  |  |  |  |  |  |  |  |
| D2 |  |  |  |  |  | 1 |  |  |  |  |  |  |  |  |
| **Dominant Hand** |  |  |  |  |  |  |  |  |  |  |  |  |  |  |
| D5 |  |  |  |  |  | 2 |  |  |  |  |  |  |  |  |
| D3 | 1 |  |  | 1 |  |  | 1 |  |  | 1 |  |  |  | 1 |
| D2 |  |  | 1 |  |  | 3 |  |  |  |  |  |  | 1 |  |
| **Total** | 1 | 1 | 1 | 1 | 1 | 25 | 1 | 1 | 1 | 1 | 1 | 1 | 2 | 1 |
